# Dietary glutamine supplementation suppresses epigenetically-activated oncogenic pathways to inhibit melanoma tumour growth

**DOI:** 10.1038/s41467-020-17181-w

**Published:** 2020-07-03

**Authors:** Mari B. Ishak Gabra, Ying Yang, Haiqing Li, Parijat Senapati, Eric A. Hanse, Xazmin H. Lowman, Thai Q. Tran, Lishi Zhang, Linda T. Doan, Xiangdong Xu, Dustin E. Schones, David A. Fruman, Mei Kong

**Affiliations:** 1 Department of Molecular Biology and Biochemistry; School of Biological Sciences, University of California, Irvine, Irvine, CA 92697 USA; 2 Center for Informatics, City of Hope National Medical Center, Duarte, CA 91010 USA; 3 Department of Computational & Quantitative Medicine, Beckman Research Institute, City of Hope Medical Center, Duarte, CA 91010 USA; 4 Department of Diabetes and Metabolic Disease, Beckman Research Institute of City of Hope Cancer Center, Duarte, CA 91010 USA; 5 Institute for Clinical and Translational Science, University of California, Irvine, CA 92687 USA; 6 UCI Health Dermatology Center, Irvine, CA 92697 USA; 7 Department of Pathology, University of California San Diego, La Jolla, CA 92093 USA

**Keywords:** Cancer metabolism, Skin cancer

## Abstract

Tumour cells adapt to nutrient deprivation in vivo, yet strategies targeting the nutrient poor microenvironment remain unexplored. In melanoma, tumour cells often experience low glutamine levels, which promote cell dedifferentiation. Here, we show that dietary glutamine supplementation significantly inhibits melanoma tumour growth, prolongs survival in a transgenic melanoma mouse model, and increases sensitivity to a BRAF inhibitor. Metabolomic analysis reveals that dietary uptake of glutamine effectively increases the concentration of glutamine in tumours and its downstream metabolite, αKG, without increasing biosynthetic intermediates necessary for cell proliferation. Mechanistically, we find that glutamine supplementation uniformly alters the transcriptome in tumours. Our data further demonstrate that increase in intra-tumoural αKG concentration drives hypomethylation of H3K4me3, thereby suppressing epigenetically-activated oncogenic pathways in melanoma. Therefore, our findings provide evidence that glutamine supplementation can serve as a potential dietary intervention to block melanoma tumour growth and sensitize tumours to targeted therapy via epigenetic reprogramming.

## Abstract (web summary)

The availability of nutrients within the tumour microenvironment can influence tumour growth. Here, the authors find that, in a melanoma mouse model, mice fed a high glutamine diet have reduced tumour growth, increased sensitivity to Braf inhibitors and elevated a-ketoglutarate levels.

## Introduction

Despite the fact that glutamine is the most abundant amino acid in serum, several studies indicate that solid tumours, such as melanoma, pancreatic cancer, and colorectal cancer, reside in a glutamine-deprived microenvironment^1–3^. As a result, tumour cells must adapt to glutamine deprivation for tumorigenesis to persist. For instance, tumour cells can undergo metabolic reprogramming to utilize other carbon sources, like asparagine and aspartate, to promote cell survival^4–6^. In melanoma, low glutamine in the tumour core led to the depletion of α-ketoglutarate (αKG), a key cofactor for the Jumonji-domain-containing histone demethylases (JHDMs), which catalyse the removal of methyl marks from histones^1,7^. This low glutamine-induced histone hypermethylation promoted melanoma tumour dedifferentiation and resistance to BRAF inhibitors. These results suggest that low glutamine in the tumour microenvironment, similar to hypoxia, may drive cancer progression and augment resistance to treatment via epigenetic regulation. However, whether increased glutamine levels in vivo can be detrimental to melanoma cells, which are well-adapted to a low glutamine environment, remains unknown.

In vitro conditioned cancer cells convert glucose to lactate and rely on glutamine to replenish tricarboxylic acid (TCA) cycle intermediates^8^. However, in contrast to previous in vitro results, accumulating evidence from in vivo experiments demonstrate that glutamine is not an essential nutritional source to support tumour growth. For instance, in vivo isotopic labelling studies, in lung cancer patients and animal models, indicated minimal utilization of glutamine in the TCA cycle with glucose and lactate identified as the main contributors to TCA intermediates^9,10^. Similarly, human glioblastoma cells relied more on glucose to support anapleorotic and biosynthetic pathways and led to accumulation of glucose-derived glutamine in the tumours in vivo^11^. Furthermore, as in vitro conditions rely on the use of culturing medium with excess nutrients, a recent study used a modified medium, in combination with hypoxia, to reproduce the in vivo metabolic environment and demonstrate that an increase in glutamine consumption does not predict its essentiality for growth^12^. Therefore, a more objective evaluation of how glutamine can affect tumour growth in vivo is needed.

Here, using several patient-derived melanoma tumour models, we found that glutamine supplementation suppresses epigenetically activated oncogenic pathways and inhibits melanoma tumour growth. Importantly, dietary glutamine supplementation increases survival in a transgenic melanoma model and improves therapeutic response to BRAF inhibition. Our work highlights the importance of understanding the in vivo effect of glutamine on melanoma tumour growth for better therapeutic strategies targeting the nutrient-poor tumour microenvironment.

## Results

### Glutamine supplementation inhibits melanoma tumour growth

**Fig. 1:**
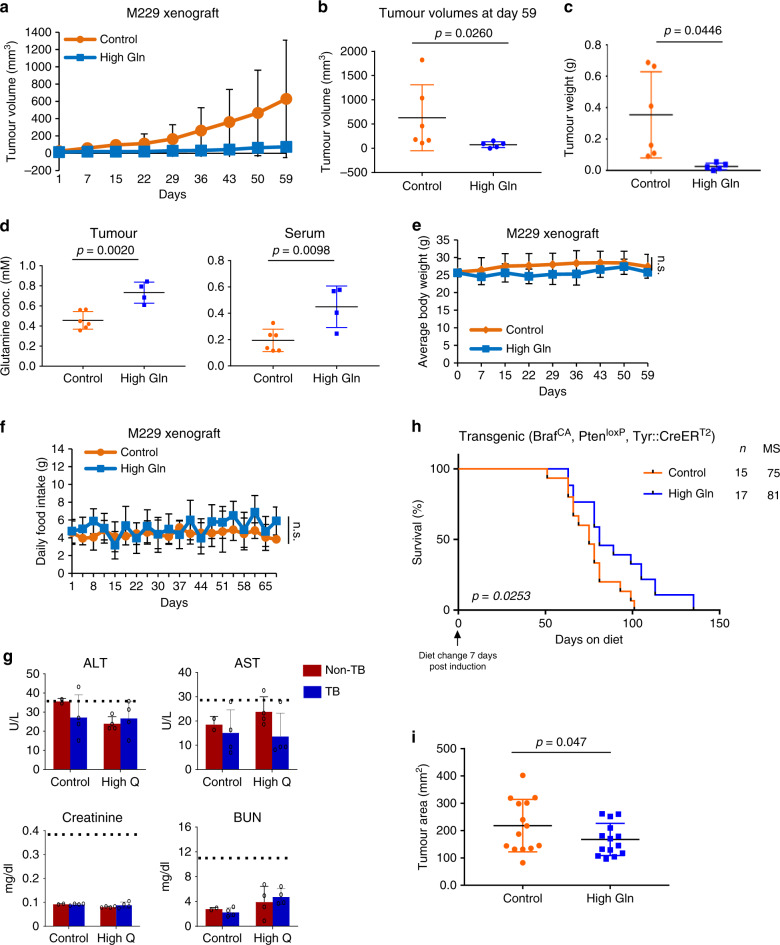
Glutamine supplementation inhibits melanoma tumour growth. **a** Nude mice with subcutaneous injection of M229 cells received control or high glutamine (High Gln) diet 1 week post injection. Tumours were measured twice weekly (Control, *n* = 6; High Gln, *n* = 5). **b** Final tumour volumes at day 59 in the control and High Gln diet groups (Control, *n* = 6; High Gln, *n* = 5 biologically independent tumours). **c** M229 xenograft tumour weights post mortem (Control, *n* = 6; High Gln, *n* = 5 biologically independent tumours). **d** Glutamine concentration from M229 tumours and nude mice serum measured by EIA kit (*n* = 6 for Control and *n* = 4 for High Gln biological replicates). **e**, **f** Body weight in **e** and food intake in **f** of nude mice injected with M229 xenografts (Control, *n* = 6; High Gln, *n* = 5). **g** Serum from M229 tumour-bearing (TB) and non-TB nude mice fed control or glutamine-supplemented (High Gln) diet. Blood was collected by cardiac puncture and analysed for liver and kidney function by EIA kit. Dashed lines represent expected levels in nude mice (Control non-TB *n* = 2, Control TB *n* = 4, High Gln non-TB *n* = 4, High Gln TB *n* = 4, biological replicates performed in triplicates). **h** B6.BRaf^CA^, Pten^loxP^, Tyr::Cre^ERT2^ tri-allelic transgenic mice placed on control or glutamine-supplemented (High Gln) diet 1 week post 4-hydroxytamoxifen topical administration until clinical end point. *p* value calculated by Mantel–Cox test. **i** B6.BRaf^CA^, Pten^loxP^, Tyr::Cre^ERT2^ tumour areas post mortem (Control, *n* = 6; High Gln, *n* = 5 biologically independent tumours). Data represent means and error bars are s.d. *p* value calculated by *t* test (unpaired, two tailed) except in **b** by Mann–Whitney test (unpaired, one tailed). n.s. not significant.

To test the effect of glutamine supplementation on melanoma tumour growth, we designed an open standard “control” diet with 16 %kcal fat and added crystalline amino acids, except for glutamine. Then we used the same diet composition supplemented with 20% glutamine compared to control diet (Supplementary Table 1). We based the diet composition on previous clinical studies using up to 30 g per day in patients without adverse effects and in vivo studies with 20–30% glutamine supplementation in rodent diet^13–15^. Mice were placed on high glutamine diet 1 week after subcutaneous xenograft injections of the patient-derived M229 cells harbouring a BRAF V600E mutation and a phosphatase and tensin homologue (PTEN) deletion. Interestingly, we found that tumour growth was significantly suppressed in mice under high glutamine diet compared to the control diet groups (Fig. 1a–c)^16^. Using a linear mixed model, we observed a difference in growth rate of 70 mm^3^ per week (95% confidence interval: 48–92, *p* < 0.001) between the two diet groups. Moreover, supplementation of glutamine in the diet significantly increased the concentration of glutamine in tumours and serum (Fig. 1d), without any significant difference in body weight, food intake, or liver and kidney physiological functions (Fig. 1e–g). Since mutant NRAS is known to drive glutamine dependency in melanoma^17^, we investigated the effect of glutamine supplementation in HMCB xenografts, which express wild-type BRAF and mutant NRAS, and found that increased glutamine intake hindered growth in these tumours compared to the control diet group (Supplementary Fig. 1a, b). Notably, in all our xenograft experiments, we observed a high variability in tumour growth using control diet compared to regular chow independent of xenograft cell injections. Despite this, these results demonstrate a significant change in tumour growth rate and volume with glutamine supplementation. To further test glutamine supplementation on syngeneic mice melanoma models, we used B16 xenografts and observed a similar confounding inhibitory effect on tumour growth (Supplementary Fig. 1c). Moreover, we sought to test whether increased dietary glutamine can extend the survival rate of tumour-bearing mice in a spontaneous and more aggressive mouse melanoma model. Thus B6.BRaf^CA^, Pten^loxP^, Tyr::Cre^ERT2^ tri-allelic transgenic mice were placed on control or glutamine-supplemented diet 1 week after administering 4-hydroxytamoxifen (4-OHT) to induce melanoma tumours^18^. We found that dietary glutamine supplementation extended the survival in these mice and reduced overall tumour burden (Fig. 1h, i).

### Increased glutamine is sufficient to inhibit melanoma growth

**Fig. 2:**
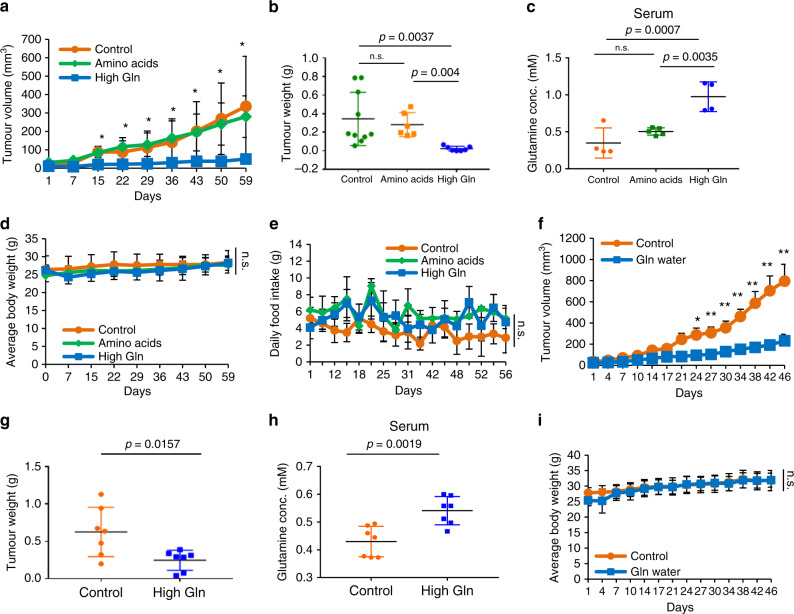
Increased glutamine is sufficient to inhibit melanoma growth. **a** Nude mice with M229 xenografts received control, amino acids, or high Gln diet. Tumours were measured twice weekly (Control, *n* = 10; Amino acids, *n* = 6; High Gln, *n* = 7). **b** Tumour weights post mortem (Control, *n* = 10; Amino acids, *n* = 6; High Gln, *n* = 7 biologically independent tumours). **c** Glutamine concentration in serum measured by EIA Kit (Control, *n* = 4; Amino acids, *n* = 5; High Gln, *n* = 4 biological replicates performed in triplicates). **d**, **e** Nude mice with subcutaneous injection of M229 cells receiving control, amino acids, or High Gln diet were weighed in **d** and monitored for food intake in **e** (Control, *n* = 10; Amino acids, *n* = 6; High Gln, *n* = 7). **f** Nude mice with subcutaneous injection of M229 cells placed on regular (control) water or glutamine-supplemented (Gln) water 1 week post injection. Tumours were measured twice weekly (Control, *n* = 7; High Gln, *n* = 7). **g** Tumour weights post mortem (Control, *n* = 7; High Gln, *n* = 7 biologically independent tumours). **h** Glutamine concentration in serum was measured (Control, *n* = 7; High Gln, *n* = 7 biological replicates performed in triplicates). **i** Nude mice with subcutaneous injection of M229 cells placed on control water or Gln water were weighed twice weekly (Control, *n* = 7; High Gln, *n* = 7 biologically independent mice). Data represent means and error bars are s.d. **a**–**e** *p* value calculated using one-way ANOVA followed by post hoc Tukey’s HSD test. **f**–**i** *p* value calculated by *t* test (unpaired, two tailed), *p < 0.05, **p < 0.01, n.s. not significant.

To maintain isocaloric intake, glutamine supplementation in diet resulted in a 20% reduction in carbohydrate content (Supplementary Table 1). Since low carbohydrate diet has previously been shown to affect growth in prostate cancer, we sought to confirm that the observed effect on tumour growth was specific to glutamine supplementation and not the reduction in dietary carbohydrates^19^. Thus we designed another control diet to replace the 20% glutamine increase with equal increase in all purified amino acids present in rodent diet (labelled Amino acids diet) while maintaining equal percentage of carbohydrates (Supplementary Table 1). Consistently, high glutamine in the diet led to significant reduction in M229 xenograft tumour volumes. Importantly, increasing total amino acids to 20% in the diet did not have an effect on tumour growth (Fig. 2a, b), yet a dose-dependent effect of individual amino acid on tumour growth remains to be determined. These results suggest that the observed change in melanoma tumour growth is specific to the increased glutamine, and not the decrease in the kcal percentage of carbohydrate, in the diet. Notably, serum glutamine levels were significantly increased only by glutamine supplementation in the diet, and no difference in body weight or food intake was observed between the diet groups (Fig. 2c–e). We also found that glutamine supplementation in drinking water was as effective in deterring melanoma tumour growth as dietary glutamine intake (Fig. 2f–i). Together, these data demonstrate that glutamine supplementation via food or water is sufficient to inhibit melanoma tumour growth.

### High glutamine impedes growth independent of BRAF status

**Fig. 3:**
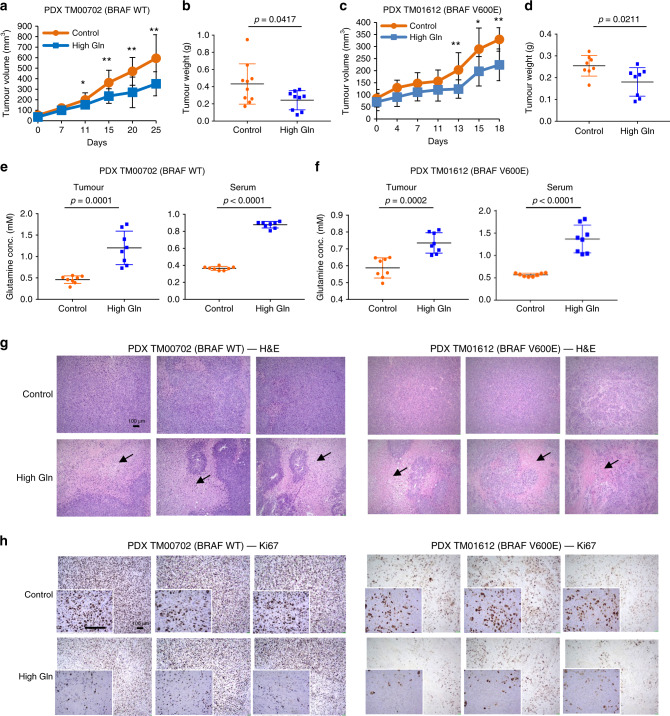
High glutamine impedes growth independent of BRAF status. **a**, **b** NSG mice with subcutaneous implantation of melanoma PDX tumour TM00702 (Control, *n* = 10; High Gln, *n* = 9 biologically independent tumours) received control or high glutamine (High Gln) diet and tumours were measured twice weekly (**a**) and tumour weights post mortem are shown in **b**. **c**, **d** NSG mice with subcutaneous implantation of melanoma PDX tumour TM01612 (Control, *n* = 8; High Gln, *n* = 8 biologically independent tumours) received control or High Gln diet and tumours were measured twice weekly (**c**) and tumour weights post mortem are shown in **d**. **e**, **f** Glutamine concentration in PDX TM00702 in **e** and TM01612 in **f** from serum and tumours were measured (*n* = 8 biological replicates). **g** Cross-sections of PDX_TM00702 (left) and PDX_TM01612 (right) tumours were H&E stained. Scale bar = 100 μm. Arrows indicate regions of necrosis/apoptosis. **h** Cross-sections of PDX_TM00702 (left) and PDX_TM01612 (right) tumours were Ki67 stained (Control, *n* = 3; High Gln, *n* = 3). Scale bar = 100 μm. Data represent means and error bars are s.d. *p* value was calculated by *t* test (unpaired, two tailed) except in **a**, **c** by Mann–Whitney test (unpaired, two tailed). *p < 0.05, **p < 0.01.

To further test the effect of increased dietary glutamine, we used two melanoma patient-derived xenograft (PDX) models with different genetic backgrounds, PDX_TM00702 (with wild-type BRAF) and PDX_TM01612 (with BRAF V600E mutation), and placed mice on either control or glutamine-rich diet when tumours reached an average of 50 mm^3^. Consistently, we found that dietary glutamine supplementation significantly reduced tumour growth in both melanoma PDX models independent of BRAF status (Fig. 3a–d). We did not observe any significant difference in body weight or food intake in mice with PDX_TM00702 tumours (Supplementary Fig. 2a, b). However, mice with PDX_TM01612 tumours exhibited weight loss in both dietary groups with no difference in food intake (Supplementary Fig. 2c, d). Serum and tumour tissues from both models showed increased glutamine levels in mice receiving the experimental diet (Fig. 3e, f), while having no significant effect on other amino acids (Supplementary Fig. 3). In addition to the marked difference in tumour volumes, cross-sections of PDX tumours from mice fed with glutamine-supplemented diet appeared to have more necrotic or apoptotic regions by haematoxylin and eosin (H&E) staining (Fig. 3g) accompanied by decreased proliferation by Ki67 staining compared to control (Fig. 3h and Supplementary Fig. 2e).

### High glutamine downregulates melanoma-associated oncogenes

**Fig. 4:**
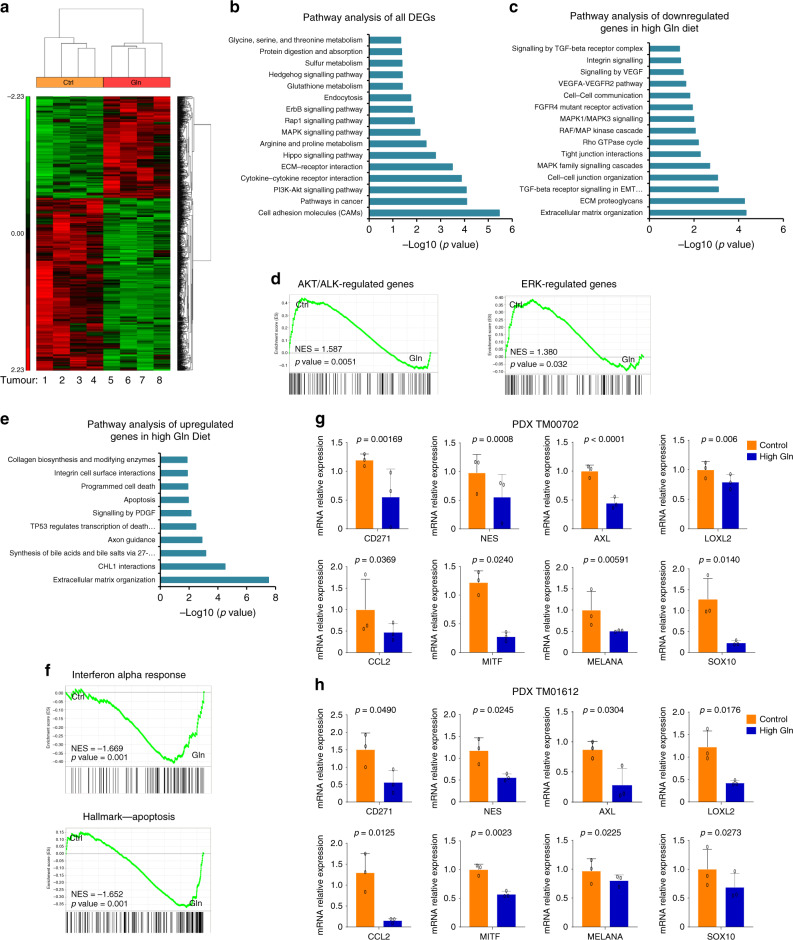
High glutamine downregulates melanoma-associated oncogenes. **a** Hierarchical clustering and heat map of differentially expressed genes (DEGs) in PDX_TM00702 tumours from control (Ctrl) or glutamine supplementation (Gln) mice groups (*n* = 4, biological replicates). **b** Pathway enrichment analysis of all DEGs (*p* < 0.05 and FDR < 0.05) in Gln versus Ctrl groups with fold change = ±1.5. **c** Pathway enrichment analysis of DEGs downregulated in Gln versus Ctrl groups with fold change ≥1.5, *p* < 0.05 and FDR < 0.05. **d** GSEA analysis in control (Ctrl) versus glutamine supplemented (Gln) tumours. **e** Pathway enrichment analysis of DEGs upregulated in Gln versus Ctrl groups with fold change <−1.5, *p* < 0.05 and FDR < 0.05. **f** GSEA analysis in control (Ctrl) versus glutamine supplemented (Gln) tumours. **g**, **h** Melanoma-associated oncogenes expression in PDX_TM00702 in **g** and PDX_TM01612 in **h** tumours verified by qPCR (*n* = 3, biological replicates performed in triplicates). Data represent means and error bars are s.d. *p* value calculated by *t* test (unpaired, two tailed). Source data are provided as a Source data file.

Low glutamine levels in tumour regions have been shown to induce upregulation of melanoma dedifferentiation genes via the inhibition of the αKG-dependent JHDMs^1^. We tested whether dietary glutamine supplementation is affecting growth through transcriptomic alterations by performing RNA-sequencing (RNA-seq) analysis in PDX melanoma tumours. We compared differentially expressed genes (DEGs) coupled with pathway enrichment and gene set enrichment analysis (GSEA) in control versus high glutamine tumours. From DEG analysis, we observed a significant and uniform change in the gene expression profile in all tumours from glutamine-fed mice with an observed overall downregulation of genes as a result of high glutamine levels (Fig. 4a and Source data). Broad pathway enrichment analyses revealed several pathways impacted by glutamine supplementation (Fig. 4b). Specifically, significantly downregulated gene sets (*p* < 0.05, false discovery rate (FDR) < 0.05) indicated a downregulation in major oncogenic pathways in mice groups receiving dietary glutamine supplementation (Fig. 4c). Moreover, GSEA analyses demonstrate that control tumours, but not tumours from mice receiving glutamine, have stronger enrichments for AKT and extracellular signal–regulated kinase (ERK)-related genes, both of which are major melanoma oncogenic signalling pathways (Fig. 4d)^20^. In comparison, upregulated genes (*p* < 0.05, FDR < 0.05) in tumours from mice receiving glutamine supplementation show significant changes in pathways related to apoptosis regulation (Fig. 4e, f and Source data). Furthermore, quantitative real-time PCR (qPCR) analysis of melanoma oncogenic genes (CD271, NES, AXL, LOXL2, MITF, MELANA, SOX10) and the melanoma chemotactic gene, CCL2, showed significant downregulation and a change in downstream signalling of these genes by glutamine supplementation in both PDX tumour models (Fig. 4g, h and Supplementary Fig. 2f)^21,22^. These data indicate that glutamine supplementation suppresses tumour growth via global repression of melanoma oncogenic genes.

### Dietary glutamine increases αKG in vivo

**Fig. 5:**
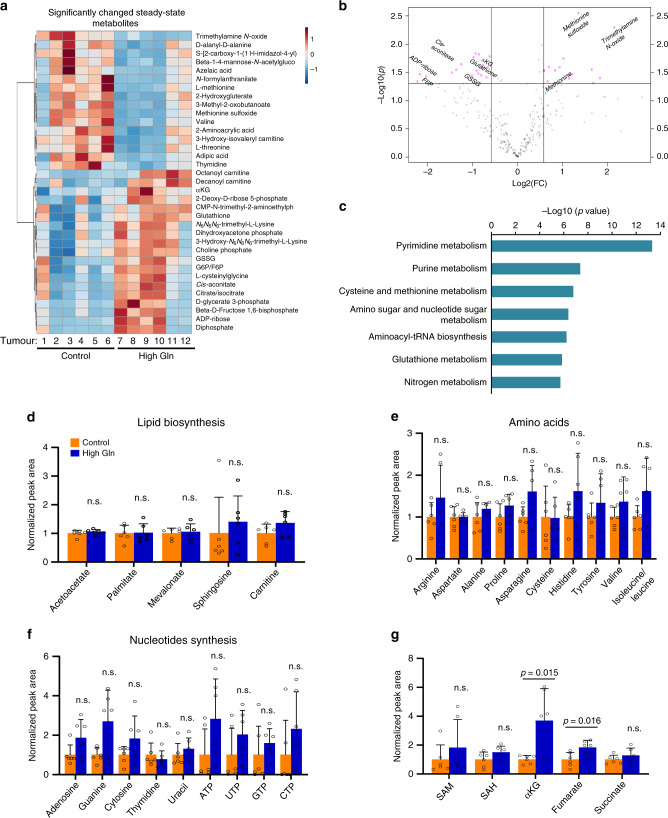
Dietary glutamine increases αKG in vivo. **a** Heat map of metabolites measured by LC-MS in PDX_TM00702 tumours (*p* < 0.05, *n* = 6, biological replicates in each group). **b** Volcano plot of significantly (represented in pink-coloured dots) changed metabolites. **c** Pathway analysis on significantly changed (*p* < 0.05 and FDR < 0.05) metabolites in control versus high glutamine PDX_TM00702 tumours. **d**–**f** Peak area of the selected metabolites related to lipid biosynthesis in **d**, amino acids in **e** and nucleotide synthesis in **f** normalized to control tumours (Control, *n* = 6; High Gln, *n* = 6 biologically independent tumours). **g** Peak area of the selected epigenetic-related metabolites normalized to control tumours (Control, *n* = 6; High Gln, *n* = 6 biologically independent tumours). Data represent means and error bars are s.d. *p* value calculated by *t* test (unpaired, two tailed). n.s. not significant. Source data are provided as a Source data file.

We next analysed metabolites from tumour tissues by liquid chromatography–mass spectrometry (LC-MS) to evaluate changes in tumour metabolism as a result of the high glutamine diet. As expected, glutamine supplementation resulted in significant changes in several metabolites, particularly in metabolites downstream of glutaminolysis including αKG (Fig. 5a, b and Source data). Pathway analysis of significantly changed metabolites (*p* < 0.05, FDR < 0.05) indicated an increase in pathways directly linked to glutamine, such as nucleotides and glutathione metabolism, in tumours from mice receiving glutamine supplementation (Fig. 5c and Supplementary Table 2). Despite the increase in nucleotide metabolism, tumours from mice supplemented with glutamine were not rapidly proliferating or increasing in size (Fig. 3a, h). Thus, to further confirm that glutamine supplementation is not contributing to biosynthetic pathways necessary for cell proliferation in vivo, we evaluated levels of cellular substrates for lipid, amino acid, and nucleotide biosynthesis^23,24^. The metabolite profile of each of these pathways revealed no significant change in their levels as a result of increased glutamine (Fig. 5d–f). Alternatively, glutamine supplementation could be inhibiting melanoma tumour growth through metabolite-driven gene expression based on the change observed in the transcriptomic profile of these tumours (Fig. 4a). This is consistent with previous studies linking glutamine levels to gene expression via αKG-mediated epigenetic reprogramming^25,26^. Thus, we examined all metabolites that were previously linked to epigenetic modifications, such as *S*-adenosyl methionine (SAM), *S*-adenosylhomocysteine (SAH), and the TCA cycle intermediates αKG, fumarate, and succinate^27–30^. We found αKG is the most increased metabolite in tumours from mice fed with high glutamine diet (Fig. 5g and Supplementary Fig. 4a). These results suggest that significant increase in αKG could be mediating transcriptomic changes as a result of the increase in glutamine in the tumour microenvironment.

### High glutamine decreases H3K4me3 methylation

**Fig. 6:**
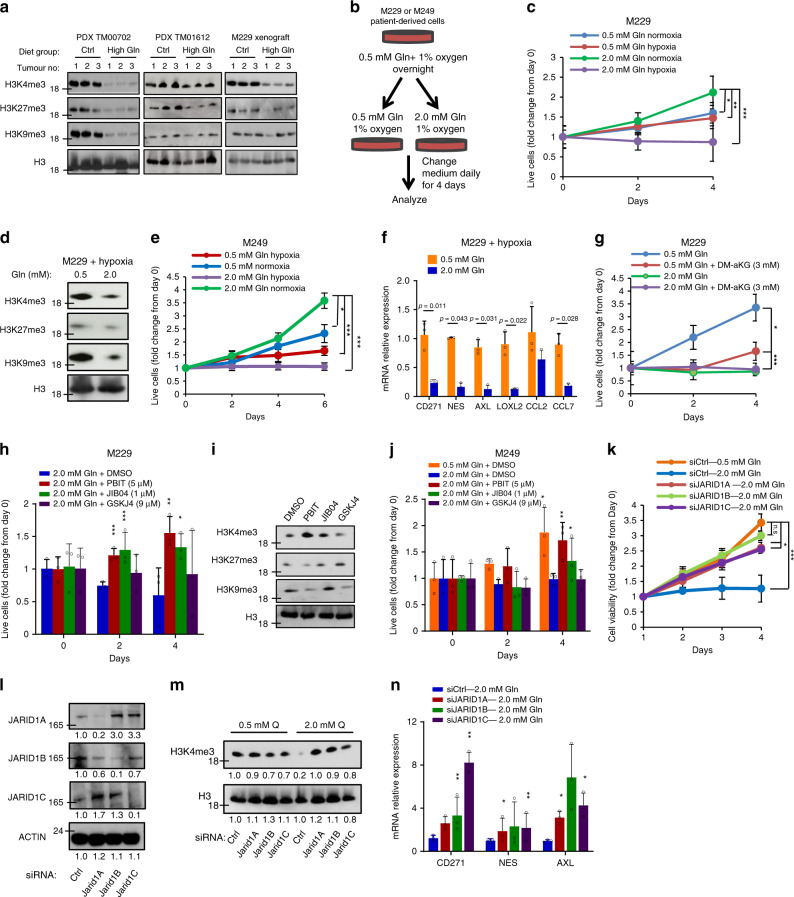
High glutamine decreases H3K4me3 methylation. **a** Tumours from the control (Ctrl) or glutamine-supplemented (High Gln) groups were used for histone extraction and assessed by western blotting using the indicated histone antibodies. **b** Schematic of the modified cell-culturing conditions in vitro to mimic tumour microenvironment. **c** M229 cells cultured in 96-well plate as in **b**. Cell viability was assessed using CellTiter Glo assay (*n* = 3, independent experiments). **d** M229 cells, cultured as in **b**, were lysed and used for histone extraction and immunoblotting with the indicated antibodies. **e** M249 cells, cultured as in **b**, were assessed using Trypan blue exclusion (*n* = 3, independent cell cultures). **f** M229 cells, cultured as in **b**, were used for qPCR analysis (*n* = 3, independent cell cultures). **g** M229 cells cultured as in **b** with or without 3 mM DM-αKG. Cell viability was assessed using CellTiter Glo assay (*n* = 3, independent cell cultures). **h**, **i** M229 cells cultured as in **b** and treated with DMSO, PBIT (5 μM), JIB04 (1 μM) or GSKJ4 (9 μM). Cells were assessed for viability by CellTiter Glo assay in **h** (*n* = 3, independent experiments) or collected for western blotting using the indicated antibodies in **i**. **j** M249 cells cultured as in **h**. Live cell numbers were assessed using Trypan blue exclusion (*n* = 3, independent experiments). **k**, **l** M229 cells were transfected with siRNA against scramble control (siCtrl), JARID1A, JARID1B or JARID1C and cultured as in **b**. Live cell numbers were assessed using Trypan blue exclusion in **k** (*n* = 3, independent cell cultures) or collected for immunoblotting as indicated in **l**. **m** siCtrl, siJARID1A, siJARID1B or siJARID1C were collected for histone extraction and immunoblotting. Immunoblots were quantified using ImageJ. **n** siCtrl, siJARID1A, siJARID1B or siJARID1C cells cultured as in **b** were collected for qPCR analysis (*n* = 3, independent cell cultures). All immunoblots are representative of three independent experiments. Data represent means and error bars are s.d. *p* value calculated by *t* test (unpaired, two tailed). *p < 0.05, **p < 0.01, ***p < 0.001. Source data are provided as a Source data file.

αKG is a necessary cofactor for several JHDM enzymes that mediate the demethylation of histone H3 on lys4, lys27, and lys9 thereby affecting access of transcriptional machinery to gene loci^25,26,31^. Histone immunoblots from PDX_TM00702, PDX_TM01612, and M229 xenograft tumours revealed a decrease in H3K4me3, H3K27me3, and H3K9me3 levels as a result of glutamine supplementation (Fig. 6a). Notably, only the decrease in H3K4me3 was consistent across all tumour models. To further investigate whether high glutamine-induced melanoma growth inhibition is mediated by histone hypomethylation, we cultured two parental patient-derived melanoma cells, M229 and M249, in tumour-like conditions, under low glutamine (0.5 mM) and 1% oxygen. We then proceeded to switch the medium to either low glutamine (0.5 mM) or high glutamine (2.0 mM), under hypoxic conditions, with the medium changed daily (Fig. 6b). Both M229 and M249 cells cultured under normoxia had increased rate of proliferation with higher glutamine concentration, in agreement with previous in vitro studies^1^. However, we found that, using these patient-derived cells under more physiological tumour oxygen levels, increased glutamine dramatically reduced cellular proliferation (Fig. 6c, e). This is in line with several studies indicating that non-physiological culturing conditions can skew cancer cell metabolism^12,32^. In addition, reduced histone methylation was observed in cells cultured with high glutamine under hypoxia, similar to the effect seen in melanoma tumours in vivo (Fig. 6d). As seen in the PDX tumours, the expression of melanoma-associated genes was also decreased in M229 cells cultured with high glutamine under hypoxia (Fig. 6f). Moreover, to further confirm that the effect of high glutamine, under hypoxia, on viability is mediated through the increase in αKG-dependent demethylation of histones, we cultured cells in different glutamine concentrations with the addition of dimethyl-αKG. The addition of cell-permeable dimethyl-αKG to cells cultured under low glutamine reduced cell viability and levels of H3K4me3 similar to the effect observed by high glutamine in the medium (Fig. 6g and Supplementary Fig. 4b). To further delineate which histone modification is critical in mediating the effect of glutamine supplementation, we treated cells cultured in high glutamine with JIB04 (a pan demethylase inhibitor), PBIT (an H3K4me3 demethylase inhibitor), or GSKJ4 (an H3K27me3 demethylase inhibitor) to determine whether these histone marks are necessary for the effect of high glutamine^33–35^. We found that the pan inhibitor JIB04 effectively rescued cell viability in cells cultured in high glutamine. Importantly, the H3K4me3 demethylase inhibitor PBIT treatment alone was sufficient to rescue cell viability to a similar extent as the pan inhibitor, suggesting that H3K4me3 is critical for glutamine-regulated modification of chromatin (Fig. 6h–j). In contrast, we did not observe an effect of JHDM inhibition on growth in cells cultured under low glutamine (Supplementary Fig. 4c). In line with these results, we further tested several melanoma cell lines with different genetic backgrounds. We found that the response in these cell lines (HMCB, SK-MEL-2, WM-266-4, and M249R) was tightly correlated with the decrease in H3K4me3 as a result of increased glutamine levels under hypoxia (Supplementary Fig. 4d, e). In comparison, the non-responding cell line, A375, had no change in H3K4me3 levels. PBIT specifically inhibits the JARID1 family demethylases, which include JARID1A, JARID1B, and JARID1C and mediate the demethylation of H3K4me3^33^. Consistently, we found that knockdown of these enzymes rescued melanoma cells cultured under high glutamine, while having no effect on cells cultured in low glutamine (Fig. 6k–m and Supplementary Fig. 4f). Despite the increase in JARID1A expression in JARID1B and JARID1C knockdown cells, these cells still had better survival under high glutamine and overall increase in the level of H3K4me3. Moreover, the knockdown of JARID1 demethylases induced the expression of melanoma-related oncogenes, which were suppressed by increased levels of glutamine (Fig. 6n).

### Dietary glutamine supplementation cooperates with targeted therapies

**Fig. 7:**
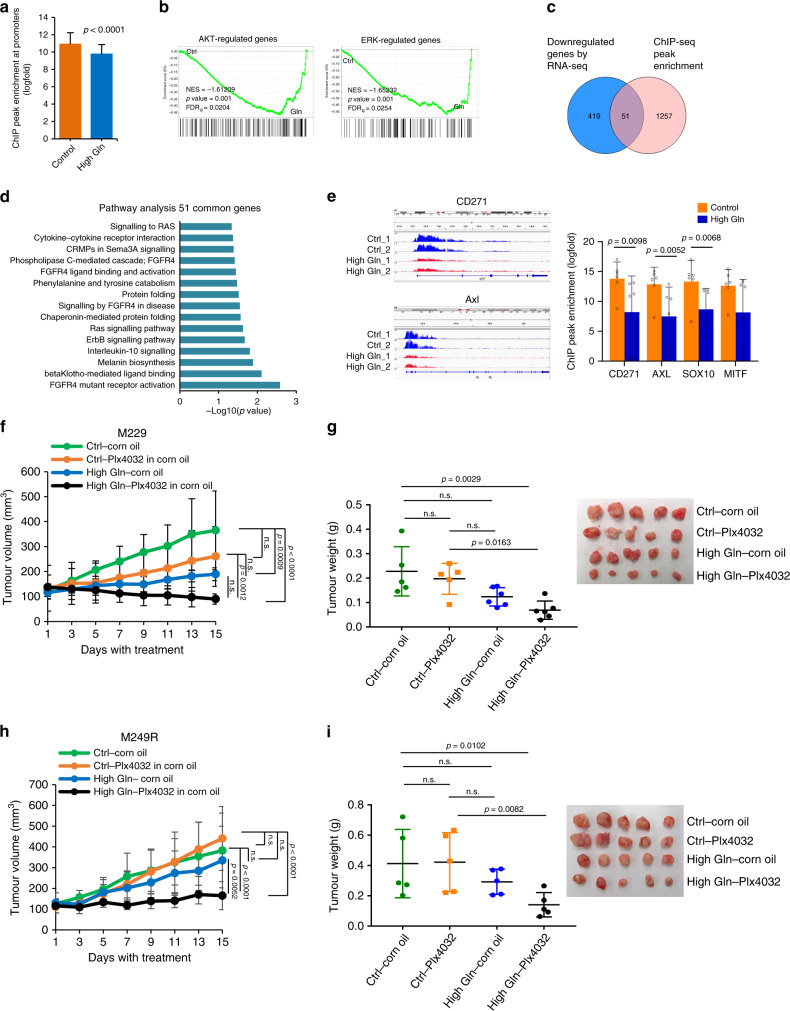
Dietary glutamine supplementation cooperates with the targeted therapies. **a** PDX_TM00702 tumours were used for ChIP-sequencing analysis using H3K4me3 antibody. ChIP peak average at the promoter regions of the associated genes (LogFC between ChIP and Input) in control and High Gln tumour samples (*n* = 3, biological replicates per group performed in technical duplicates). **b** GSEA analysis of H3K4me3 ChIP peak-associated genes in control (Ctrl) versus glutamine supplemented (Gln) tumours. **c** Venn diagram of DEGs downregulated in RNA-seq (*p* < 0.05, FDR < 0.05) and ChIP-seq gene peaks specific to promoter region in PDX-TM00702 tumours (*p* < 0.05, LogFC = 1.2). **d** Pathway analysis of 51 common genes between RNA-seq and ChIP-seq (*p* < 0.05). **e** Representation (left) and quantitation (right) of average H3K4me3 peaks at the promoter regions in melanoma-associated oncogenes (Control, *n* = 6; High Gln, *n* = 6 biologically independent tumours). **f**, **g** Nude mice with M229 xenografts received control (Ctrl) or glutamine-supplemented (High Gln) diets 1 week post injection. When tumours reached an average of 100 mm^3^ in volume, mice were treated with corn oil or 10 mg/kg PLX4032 in corn oil by oral gavage for 2 weeks. (Control-oil, *n* = 5; Control-Plx4032, *n* = 5; High Gln-oil, *n* = 6; High Gln-Plx4032, *n* = 6). Tumour volume in **f** and tumour weight in **g** are shown. **h** Nude mice with M249R xenografts received control (Ctrl) or glutamine-supplemented (High Gln) diets 1 week post injection. When tumours reached an average of 100 mm^3^ in volume, mice were treated with corn oil or 10 mg/kg PLX4032 in corn oil by oral gavage for 2 weeks (Control-oil, *n* = 5; Control-Plx4032, *n* = 5; High Gln-oil, *n* = 5; High Gln-Plx4032, *n* = 5). Tumour volume in **h** and tumour weight in **i** are shown. Data represent mean and error bars are s.d. *p* value was calculated by two-way ANOVA followed by post hoc Tukey’s multiple comparison test except in **e** by *t* test (unpaired, two tailed). n.s not significant.

As H3K4me3 is a marker for transcription initiation, we investigated whether downregulation of melanoma genes in tumours was directly linked to decreases in H3K4me3 level. We performed H3K4me3 chromatin immunoprecipitation–sequencing (ChIP-seq) and observed a significant decrease in overall H3K4me3 mapping near the promoter regions in PDX_TM00702 tumours from mice receiving glutamine supplementation (Fig. 7a and Supplementary Fig. 5). GSEA analysis indicated that glutamine supplementation affected H3K4me3 enrichment at genes involved in melanoma oncogenic pathways such as AKT and ERK, previously observed by RNA-seq analysis (Fig. 7b). Correlating downregulated DEGs by RNA-seq and genes with decreased H3K4me3 ChIP-seq enrichment at the promoter showed an overlap of 51 genes, which are involved in several oncogenic pathways such as RAS and FGFR4 signalling (Fig. 7c, d)^36,37^. Importantly, we observed a significant decrease in H3K4me3 enrichment at the melanoma-associated genes, CD271, AXL, SOX10, and MITF (Fig. 7e). These results further demonstrate that high glutamine levels in the tumour microenvironment reduce H3K4me3-dependent transcription and affect the expression of critical melanoma-associated oncogenes. Since our RNA-seq and ChIP-seq analyses indicated that increased glutamine levels repressed the global expression of melanoma-related oncogenes, we sought to test whether glutamine supplementation can cooperate with current targeted therapies. Thus we used the BRAF inhibitor, PLX4032, in M229 and PLX4032-resistant M249R xenograft tumours when tumours reached an average of 100 mm^3^ in volume^38^. Interestingly, tumours in mice fed with high glutamine diet were much more sensitive to PLX4032 treatment indicating that combining these interventions could improve therapeutic response (Fig. 7f, g). More importantly, even though glutamine supplementation had a very modest effect on tumour growth in M249R tumours, these tumours exhibited significantly slower growth when glutamine supplementation was combined with PLX4032 treatment (Fig. 7h, i). These results indicate that glutamine supplementation in diet can potentially re-sensitize tumours by downregulating mitogen-activated protein kinase and other oncogenic pathways that give rise to targeted resistance in melanoma.

## Discussion

Our results show evidence that glutamine supplementation can slow growth in melanoma tumours with different BRAF status by inhibiting epigenetically activated oncogenic pathways. Taken with previous studies on glutamine supplementation to boost the immune response without any side effects, our work highlights the potential benefit of glutamine supplementation in melanoma patients^14,15,39^. Similarly, several studies have now explored the therapeutic benefits of disrupting the tumour microenvironment in vivo. For instance, dietary serine and glycine limitation or use of ketogenic diet effectively inhibited tumour growth in several models^40–42^. In addition, supplementation with histidine or adopting a ketogenic regimen sensitized cancer to targeted treatments^43,44^. The effect of dietary intervention in treating several metabolic diseases, including cancer, has been gaining interest. Therefore, it is critical to scientifically examine how diet can serve as a molecular therapeutic tool in cancer treatments as part of a “personalized diet” for patients. Our current work focussed on melanoma, and further work is needed to determine whether other solid tumours are also sensitive to glutamine supplementation.

In vitro studies showing glutamine can be utilized by tumour cells raise a concern that glutamine will provide nutritional support to promote tumour growth in vivo. Here we found that glutamine supplementation increased several TCA intermediates; however, it was not directly utilized for growth and proliferation as indicated by Ki67 staining in melanoma tumours. Consistently, independent isotopic labelling studies in human lung cancer indicated minimum glutamine utilization in vivo, with glucose and lactate being the main contributors to the TCA cycle^9,45^. In addition, using a modified in vitro culturing system, which combines hypoxia and physiological levels of glutamine, we found that patient-derived melanoma cells exhibit differential metabolism in normoxia compared to hypoxia, indicating that hypoxia in the tumour microenvironment greatly affects glutamine metabolism. In support with this, several studies highlighted that cells experiencing hypoxia or mitochondrial dysfunction have decreased glutamine oxidation and rely on carbon sources, other than glucose and glutamine, to supply metabolic intermediates under hypoxic conditions^46–48^. These studies and others support the idea that, under low oxygen levels in the tumour microenvironment, glutamine supplementation may not increase tumour growth in vivo.

Accumulating evidence indicates that epigenetic modifications contribute to tumour progression and therapeutic responses^49^. The reversible nature of epigenetic modifications led to the emergence of novel epigenetic therapeutics, and several types of these medications have been approved for clinical use. Despite the success of these drugs in changing the epigenetics of various cancers, its efficacy in solid tumours is lagging behind. In melanoma, a solid tumour, how epigenetic reprogramming affects tumour growth is not well studied. Our data indicate that expression of several oncogenic genes in melanoma tumours are regulated at the epigenetic level, particularly, by H3K4me3. Globally increased H3K4me3 levels have been associated with metastasis and poor prognosis in cancer^50,51^. Thus a regimen that can decrease levels of H3K4me3 would be clinically beneficial. More importantly, glutamine suppression of melanoma oncogenic pathways via a global decrease in H3K4me3 levels can not only cooperate with but also re-sensitize resistant tumours to targeted therapies. These data also suggest that developing inhibitors targeting H3K4me3 methyltransferases may inhibit melanoma tumour growth and sensitize tumours to current treatments.

In addition, the availability of several metabolites has been long suspected to play a role in regulating enzymes that carry out several epigenetic modifications. Of these, αKG has been realized as a key glutamine metabolite that regulates the activity of several JHDMs^30,52^. Although the specificity of each JARID1 enzyme to particular genes or regions in the genome remains unknown, the knockdown of JARID1A, JARID1B, and JARID1C in our study directly impacted the expression of several melanoma oncogenes. Notably, we observed an increase in the expression of JARID1A by the knockdown of JARID1B or JARID1C; however, these cells still exhibited an increase in H3K4me3. Despite the fact that our results suggest that the inhibition of H3K4me3 demethylation enzymes is able to reverse the effect of high glutamine on cell growth, we are not able to exclude the potential role of these demethylases in other cellular functions.

Taken together, our results provide a previously unidentified mechanism by which glutamine supplementation inhibits melanoma tumour growth and suggests a previously unrealized therapeutic avenue using glutamine supplementation in melanoma to cooperate with current therapies and potentially combat resistance mechanisms. Furthermore, our results provide important evidence that glutamine supplementation, rather than the nutrient-limiting approaches, is a simple dietary intervention that has the potential to block melanoma tumour growth and potentiate the effects of anti-melanoma treatments by suppressing epigenetically activated oncogenic pathways.

## Methods

### Cell culture

Patient-derived melanoma M229, M249, and M249R cells were obtained from Roger S. Lo’s laboratory (UCLA)^53^. B16-OVA cells were kindly provided by Roberto Tinoco’s laboratory (UCI). HMCB, SK-MEL-2, A375, and WM-266-4 cells were purchased from ATCC. Cells were cultured at 37 °C in 5% CO_2_ in Dulbecco’s modified Eagle’s medium (DMEM) with high glucose (4.5 g/L) (DM-22, Omega, Tarzana, CA) supplemented with 10% dialysed fetal bovine serum (FBS) (FB-07, Omega, Tarzana, CA), 100 units/mL of penicillin, 100 μg/mL of streptomycin (Gemini Bio-Products, Sacramento, CA), and either 0.5 or 2.0 mM L-glutamine (GS-60, Omega, Tarzana, CA). For hypoxia culture, cells were seeded in DMEM supplemented with 10% dialysed FBS and 0.5 mM glutamine under 1% O_2_ in BioSpherix Xvivo system (Parish, NY, USA). After 24 h, the medium was changed to DMEM supplemented with 10% dialysed FBS and either 0.5 or 2.0 mM glutamine. Cells were incubated under hypoxic conditions for 4 days with the medium changed daily. Cells were tested for mycoplasma using the MycoAlert Mycoplasma Detection Kit (Lonza, Switzerland).

### Histone extraction and western blotting

Tumour tissues and cells were used for histone extraction as previously described^1^. Briefly, 3 × 10^6^ cells or 20 mg of homogenized tissues (homogenized using the Precellys Ceramic Kit with Precellys 24 homogenizer) were lysed in hypotonic lysis buffer (10 mM HEPES, 10 mM KCl, 1.5 mM MgCl_2_, 0.5 mM dithiothreitol) with protease inhibitor (Roche) on ice for 1 h. Lysate was rotated at 4 °C overnight in a final concentration of 0.2 N H_2_SO_4_. Samples were centrifuged, and supernatants were used for histone precipitation using 30% (w/v) TCA for 1 h on ice. Pellet was washed with ice-cold acetone and dissolved with water. For western blotting, cells or tumour tissues were lysed in RIPA buffer. Protein concentration was determined using the BCA Protein Assay Kit (Life Technologies, Carlsbad, CA, USA). Blots were blocked for 1 h in 5% non-fat milk in phosphate-buffered saline (PBS) with 0.05% Tween-20, and membranes were probed with primary antibodies overnight and horseradish-peroxidase-conjugated secondary antibodies (Bio-Rad, Hercules, CA, USA). Signal was visualized by Western Lightning Plus-ECL (PerkinElmer, Waltham, MA, USA). Immunoblots were quantified using ImageJ, and values were divided by actin or total H3 histones normalized to the control treatment values. The following antibodies were used: H3K4me3 (Millipore 17-614) 1:2000, H3K9me3 (Abcam ab8898) 1:1000, H3K27me3 (Millipore 17-622) 1:2000, H3 (Cell Signaling Tech 4499 monoclonal, clone# D1H2) 1:2000, JARID1A (Cell Signaling Tech 3876 monoclonal, clone# D28B10) 1:1000, JARID1B (Cell Signaling Tech 3273) 1:1000, JARID1C (Cell Signaling Tech 5361 monoclonal, clone# D29B9) 1:1000, and β-actin (Sigma A1978 monoclonal, clone# AC15) 1:10,000.

### Cell viability assays

Cells were seeded in 96- or 12-well plates and allowed to adhere overnight in 0.5 mM glutamine medium. Afterwards, medium was changed to either medium containing 0.5 or 2.0 mM glutamine. Reagents such as dimethyl sulfoxide (DMSO) and dimethyl-αKG were purchased from Sigma Aldrich (St. Louis, MO, USA) and PBIT, JIB04, and GSKJ4 were purchased from Tocris Bio-Techne (Minneapolis, MN, USA). Medium was replaced daily. Viable cell number was assessed by CellTiter Glo (Promega, Madison, WI, USA) or Trypan Blue exclusion and counted by TC20 automated cell counter (Bio-Rad, Hercules, CA, USA).

### Animal models

All studies involving animals were performed according to and approved by the Institutional Animal Care and Use Committee (IACUC) protocols at the University of California, Irvine in compliance with ethical regulations. NCr Nude mice (nu/nu, male 4 weeks old, Taconic Bioscience) were used for xenograft studies and NSG mice (NOD scid gamma, male 4 weeks old, The Jackson Laboratory) were used for PDX studies. For xenograft studies, 2 × 10^6^ or 5 × 10^6^ of M229 and M249R in 100 μL DMEM without FBS or antibiotics were injected subcutaneously into 6-week-old male NCr Nude mice. Mice were on normal chow diet for 1 week post xenograft injection. In mice melanoma model, 1 × 10^5^ B16 cells in 100 μL DMEM without FBS or antibiotics were injected subcutaneously into 6-week-old male C57BL/6 mice and placed on diets immediately. Tumour-bearing mice were placed randomly on control, glutamine-rich, or amino acid diets. For PDX studies, NSG mice were purchased from the Jackson Laboratory carrying the TM00702 or TM01612 tumours. The tumours were excised, and small pieces were implanted subcutaneously in 6-week-old male NSG mice as previously described^54^. Mice were placed randomly into 2 dietary groups 1 week post engraftment. Tumour size was measured every 2–3 days with calipers, and tumour volume was calculated using the formula ½ (length × width^2^). Mice were euthanized when tumours reached 1 cm^3^ in volume, upon ulceration/bleeding, drop in weight, or signs of lethargy. For PLX4032 treatment, when the tumour volume reached an average of 100 mm^3^, mice were placed randomly into 4 experimental groups and treated with corn oil (control) or PLX4032 (dissolved in DMSO and diluted in corn oil to 10 mg/kg) by oral gavage daily. Mice were euthanized after 2 weeks of treatment. The B6.BRaf^CA^, Pten^loxP^, Tyr::Cre^ERT2^ tri-allelic transgenic mouse model was purchased from the Jackson Laboratory (4 weeks old). Mixed male and female population were used. Activation of BRAF and *Pten* deletion were induced by topical administration of 4-OHT as previously described^18^. Mice were placed into 2 dietary groups 1 week following 4-OHT administration. All mice were monitored to determine a humane clinical endpoint. Animals that died from illnesses unrelated to tumour growth were included as censored observations. Sample sizes for each animal study were estimated based on preliminary data or previous experience with these models predicting variance within each group.

### Immunohistochemistry

After euthanization, tumours were collected and fixed in 10% formalin. Formalin-fixed, paraffin-embedded blocks of melanoma PDX tumours were used for H&E and Ki67 staining. Slides were blindly evaluated by two independent pathologists to assess the extent of necrosis or apoptosis and Ki67 staining in tumours. Ki67 microscopic images of PDX TM00702 and PDX TM01612 tumours were manually quantified to determine percentage of Ki67-positive cells.

### Diets

All mice were kept on normal chow diet until the start of the experiments. Three diets were used in this study based on the open standard diet with 16 %kcal fat with crystalline amino acids from Research Diets Inc. (New Brunswick, NJ, USA). The Control diet (A11112201) contained all essential amino acids and non-essential amino acids as specified by Research Diets. Glutamine-supplemented diet contained all amino acids equal to the control diet with the addition of 200 g of glutamine. Corn starch content was adjusted to achieve the isocaloric intake. Amino acid diet contained all amino acids equal to the control diet with a proportional increase in each amino acids to achieve the same total protein as the glutamine-rich diet. For glutamine supplementation in drinking water, the amount of glutamine-supplemented diet food intake per day was calculated to be 0.5 g per cage per day. The same amount of glutamine was dissolved in the drinking water and changed daily.

### Small interfering RNA (siRNA) cell transfection

M229 cells were transiently transfected with siRNA according to the manufacturer’s protocol by Lipofectamine RNAiMAX (Life Technologies, Carlsbad, CA, USA) with on-target SMARTpool control siRNA (D-001810-10-20) and siRNA targeting human JARID1A (KDM5A) (L-003297-02-0005), human JARID1B (KDM5B) (L-003297-02-0005), and human JARID1C (KDM5C) (L-010097-01-0005) purchased from Dharmacon (Lafayette, CO, USA). Transfected M229 cells were cultured in medium with 0.5 mM glutamine. Medium was changed to 2.0 mM glutamine after 24 h and changed daily for 4 days for cell viability or for 3 days for mRNA extraction.

### Glutamine extraction and measurement

Glutamine was extracted from 20 to 40 mg of tumour tissues homogenized in 70% ethanol by a Percellys 24 homogenizer. After spinning down cell debris, the supernatant was evaporated to dryness and dissolved in distilled water (1 μL water per mg of tissue). Glutamine concentration was determined by the EnzyChrom Glutamine Kit (EGLN-100, BioAssay Systems, Hayward, CA, USA). Whole blood was collected from mice by cardiac puncture in BD vacutainer tubes (Fisher Scientific, Hampton, NH, USA) and centrifuged for 40 min at 2000 revolutions per minute to separate serum. Non-haemolysed serum was used according to the manufacturer’s protocol.

### Physiological markers

Mouse liver functions were assessed by measuring serum alanine transaminase and aspartate transaminase (EALT-100 and EASTR-100, respectively, EnzyChrom, BioAssay Systems) according to the manufacturer’s protocol. Mouse kidney functions were assessed by measuring serum creatinine and blood urea nitrogen levels (DICT-500 and DIUR-500, respectively, Quantichrome, BioAssay Systems) according to the manufacturer’s protocol. Samples with insufficient volumes were diluted with assay buffer provided from the manufacturer. All assays were run with biological replicates in technical triplicates normalized to control levels.

### Liquid chromatography–mass spectrometry

Ten-to-15 mg of tumour tissues were cut on dry ice and soaked in pre-cooled 80% methanol in high-performance LC (HPLC)-grade water. Samples were homogenized by Precellys 24 homogenizer using the Precellys Ceramic kit. Samples were centrifuged at 4 °C for 15 min, and 500 μL of supernatant was transferred to a new tube and evaporated to dryness at room temperature (RT). For serum, 20 μL was dissolved in 80 μL ice-cold HPLC-grade water. Four hundred microlitres of cold HPLC-grade methanol were added to each sample (for a final concentration of 80% v/v). Samples were vortexed and left on ice for 10 min. After centrifugation, supernatant was transferred to new tube and evaporated to dryness using speed vacuum. The samples were prepared and analysed by LC-MS at Duke University as previously described^55^. MetaboAnalyst was used to analyse significantly changed metabolites and generate heat map, principal coordinate analysis, and volcano plot (www.metaboanalyst.ca/).

### RNA-seq library preparation

Total RNA was extracted using Trizol reagent (15596026, Life Technologies, Carlsbad, CA, USA) from tumour tissues. RNA-seq libraries were prepared with the Kapa RNA mRNA HyperPrep Kit (Kapa Biosystems, Cat KR1352) according to the manufacturer’s protocol. Briefly, 100 ng of total RNA from each sample was used for polyA RNA enrichment using magnetic oligo-dT beads. The enriched mRNA was fragmented using heat and magnesium, and the first-strand cDNA was made using random priming. The libraries were validated with the Agilent Bioanalyzer and quantified with Qubit. The RNA-seq sequence reads were mapped to *Homo sapiens* genome assembly GRCh37 (hg19) using the open source RNA-seq alignment tool HISAT2. The alignment results were converted to RNA-seq gene expression measurement as RPKM (reads/kilo base of total exon length/million mapped) using Partek Genome Suite (v6.6) and normalized to gene models in the NCBI RefSeq database. Gln samples were compared with the Control sample group using analysis of variance (ANOVA) with +/−1.5 fold change (*p* value < 0.05, FDR < 0.05) as the minimum threshold. Maximum expression <0.5 RPKM was used to filter and exclude very low expressing genes. Common genes between RNA-seq and ChIP-seq were analysed using IMPaLA version 11 pathway overrepresentation analysis.

### Sequencing with Illumina Hiseq2500

Both RNA-seq and ChIP-seq Library templates were prepared for sequencing using cBot cluster generation system (Illumina, San Diego, CA, USA) with the HiSeq SR Cluster V4 Kit. Sequencing run was performed in the single read mode of 51cycle of read1 and 7 cycles of index read using Illumina HiSeq 2500 with HiSeq SBS V4 Kits. Real-time analysis (RTA) 2.2.38 software was used to process the image analysis and base calling.

### Quantitative real-time PCR

Total RNA, from cell culture or tumour tissues, was extracted and purified using Trizol reagent (Life Technologies, Carlsbad, CA, USA) according to the manufacturer’s guidelines. One microgram of RNA was used for the qScript cDNA Synthesis Kit (Quanta Biosciences, Beverly, MA, USA). qPCR analyses were performed with SYBR Green PCR (Quanta Biosciences) using CFX Connect Real-Time PCR Detection System (Bio-Rad, Hercules, CA, USA). Expression results were normalized to β-actin levels, and all qPCR amplifications were performed in triplicates and repeated in three independent experiments. The following forward and reverse primers were generated to check gene expression: HUMAN (F: forward, R: reverse) ACTIN-F: 5′-CACCAACTGGGAGGACAT-3′ R: 5′-GCACAGCCTGGATAGCAAC-3′; CD271-F: 5′-TTGGGGGCTTGCAAGTATGT-3′ R: 5′-GTTTCAGGAGGGCCCAAGAA-3′; NES-F: 5′-CCCAACTTGGAGGGGAAGTC-3′ R: 5′-TCTCCCTCAGAGACTAGCGG-3′: AXL-F 5′-ATTCATTCCAAACCCCTGACT-3′; R: 5′-ACTGGTTAGTGATGGCCCTA-3′, LOXL2-F: 5′-CTGGTCAACAAGGCAAGAG-3′; R: 5′-AGGAATTCAGGGTCTTGCTA-3′; CCL2-F: 5′-CCAAAGAAGCTGTGATGTGA-3′; R: 5′-GCACTCTCTGACTCTAGGTTT-3′; CCL7-F: 5′-AAAATCCCTAAGCAGAGGCT-3′ R: 5′-ACCTAGGACTGAGGTGTGAG-3′; MITF-F: 5′-GATCCTATGGACTGGGCTAT-3′ R: 5′-CCAACCAAGGACATGAGAAT-3′; MELANA-F: 5′-AAGAAGGGTTTGATCATCGG-3′ R: 5′-ACAGCCATTCATGAAAATACC-3′; SOX10-F: 5′-CCAGCTAAACCCATCTGG-3′ R: 5′-CACACCAAGAGACGGTTG-3′.

### ChIP-seq library preparation

Ten milligrams of frozen tumour tissue was minced and crosslinked using 1% formaldehyde in PBS for 10 min at RT. Cross-linking was stopped following centrifugation by resuspending the pellet in 125 mM glycine in PBS for 5 min at RT. Pellets were washed twice with PBS followed by lysis in sodium dodecyl sulfate (SDS) lysis buffer [1% SDS, 10 mM ethylenediaminetetraacetic acid (EDTA), and 50 mM Tris–HCl, pH 8] supplemented with protease inhibitors (Roche). Sonication was performed using Diagenode BioruptorR pico (Leige, Belgium) for 7 cycles (30 s on/30 s off) to produce DNA fragments of 100–300 bp in length. The sonicated lysate was centrifuged and diluted using 10 volumes of cold ChIP‐dilution buffer (0.01% SDS, 1.1% Triton X‐100, 1.2 mM EDTA, 16.7 mM Tris‐HCl, pH 8, 167 mM NaCl). The pull‐down was performed with anti‐H3K4me3 antibody (Millipore Sigma, Burlington, MA, USA) and magnetic protein G Dynabeads (Cat. No. 10004D; Life Technologies, Carlsbad, CA, USA) overnight at 4 °C. Beads were washed with low salt buffer (0.1% SDS, 1% Triton X‐100, 2 mM EDTA, 20 mM Tris–HCl, pH 8, and 150 mM NaCl), high salt buffer (0.1% SDS, 1% Triton X‐100, 2 mM EDTA, 20 mM Tris–HCl, pH 8, and 500 mM NaCl), LiCl buffer (250 mM LiCl, 1% NP40, 1% NaDOC, 1 mM EDTA, and 10 mM Tris–HCl, pH 8), twice with TE buffer(10 mM Tris–HCl, pH 8, and 1 mM EDTA), and finally with elution buffer (1% SDS and 100 mM NaHCO_3_). The DNA–protein complexes were then reverse crosslinked by adding 200 mM NaCl and 20 μg Proteinase K (Sigma Aldrich, St. Louis, MO, USA) and incubated at 65 °C for 4 h. Subsequently, 20 μg of RNaseA (Sigma Aldrich) was added and further incubated for 15 min at 37 °C. The immunoprecipitated DNA was extracted using phenol/chloroform and ethanol precipitation. Resultant ChIP DNA was quantified using the Quant-iT™ dsDNA Assay Kit (Cat No. Q33120; ThermoFisher Scientific) and used for library preparation. ChIP-seq libraries were prepared with the Kapa DNA HyperPrep Kit (Cat No. KK 8700, Kapa) according to the manufacturer’s protocol. A 10 cycles of PCR was performed to produce the final sequencing library. The libraries were validated with the Agilent Bioanalyser DNA High Sensitivity Kit and quantified with Qubit. The ChiP-Seq sequence reads were mapped to *H. sapiens* genome assembly GRCh37 (hg19) using open source DNA-seq alignment tool BWA (v). High-quality unique alignments were used with SAM Tag (XA:Z and SA:Z) as filter and removed the duplicated alignments caused by PCR duplication using SAMTools (ver 0.1.19). The unmapped reads were removed using SAMTools based on the alignment quality tag (-F 4 -F 256). The cross-correlation of the reads aligned to both strands was tested as ChiP QC using R’s SPP package (v1.13) before ChIP-seq peak calling using MACS2 (v2.1.2) with broad option to detect the H3K4me3-binding sites with FDR < 0.005 cutoff.

### GSEA analysis

GSEA was performed against hallmarks and oncogenic signature gene set MSigDB database v6.2 database with data set ranked from LogFC (http://software.broadinstitute.org/gsea/msigdb).

### Statistics

All in vitro experiments were repeated three independent times with at least technical triplicates. Results are shown as means; error bars represent standard deviation (s.d.) as specified in figure legends. Unpaired *t* test and one-way or two-way ANOVA followed by post hoc test were used to determine the statistical significance of differences between means and were calculated in the Microsoft Excel or GraphPad Prism (v7) software. When determining the effect of high glutamine diet using control versus test groups, a linear mixed model (multiple comparisons) and Mann–Whitney test were used to determine *p* value. When two dietary groups were compared to control diet, a linear mixed model and one-way ANOVA followed by Tukey’s Honestly Significant Difference test was used to determine *p* values. A multiple comparison fitting a mixed model two-way ANOVA was applied when comparing two factors, such as diet and BRAF-targeting agent on tumour growth. Statistical comparison of the survival curves was calculated using the GraphPad Prism (v7) software using Mantel–Cox (Log Ranks) test. (*p < 0.05, **p < 0.01, ***p < 0.001, unless indicated separately).

### Reporting summary

Further information on research design is available in the Nature Research Reporting Summary linked to this article.

## Supplementary information

Supplementary Information

Reporting Summary

## Data Availability

Data that support the findings of this study have been deposited in the Gene Expression Omnibus (GEO) under the accession codes: ChIP-seq data, GSE125822 and RNA-seq data, GSE140274. The source data underlying Figs. 1, 2, 4a–e, 5 and 6 and Supplementary Fig. 4 are provided as a Source data file. All other data sets are available within the article and Supplementary Information or available from the corresponding author upon request. Source data are provided with this paper.

## Source data

Source Data
