# Supplementary material for: Dietary glutamine supplementation suppresses epigenetically-activated oncogenic pathways to inhibit melanoma tumour growth
Source: Nat Commun. 2020 Jul 3;11:3326. doi: 10.1038/s41467-020-17181-w (PMC7335172; doi:10.1038/s41467-020-17181-w)
Supplement: Supplementary file 1 — Supplementary Information [file 41467_2020_17181_MOESM1_ESM.pdf]

Supplementary Information

Dietary glutamine supplementation suppresses epigenetically-activated oncogenic pathways to inhibit melanoma tumour growth

Ishak Gabra et al.

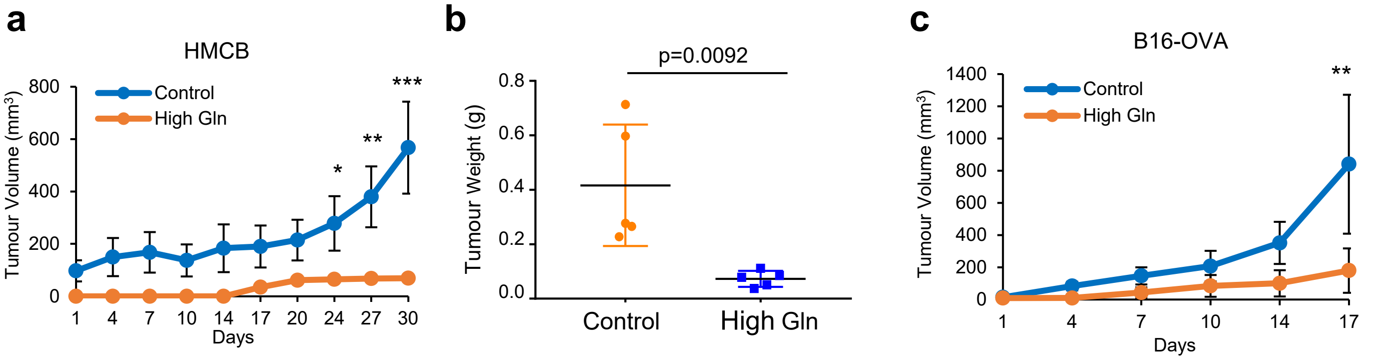

Supplementary Figure 1. a. Nude mice with subcutaneous injection of HMCB cells received control or high glutamine (High Gln) diet one-week post injection. Tumours were measured twice weekly from day 1 (when tumours are visible). (Control, n=5; High Gln, n=5). **b.** HMCB xenograft tumour weights post-mortem**.** (Control, n=5; High Gln, n=5 independent tumours). **c.** B16 cells were injected subcutaneously in C57BL/6 mice and randomly placed on control or high glutamine (High Gln) diet post injection. Tumours were measured twice weekly (Control, n=6; High Gln, n=6). Data represent means. Error bars are s.e.m. p value calculated by t-test (unpaired, two-tailed). *p<0.05, **p<0.01, ***p<0.001.

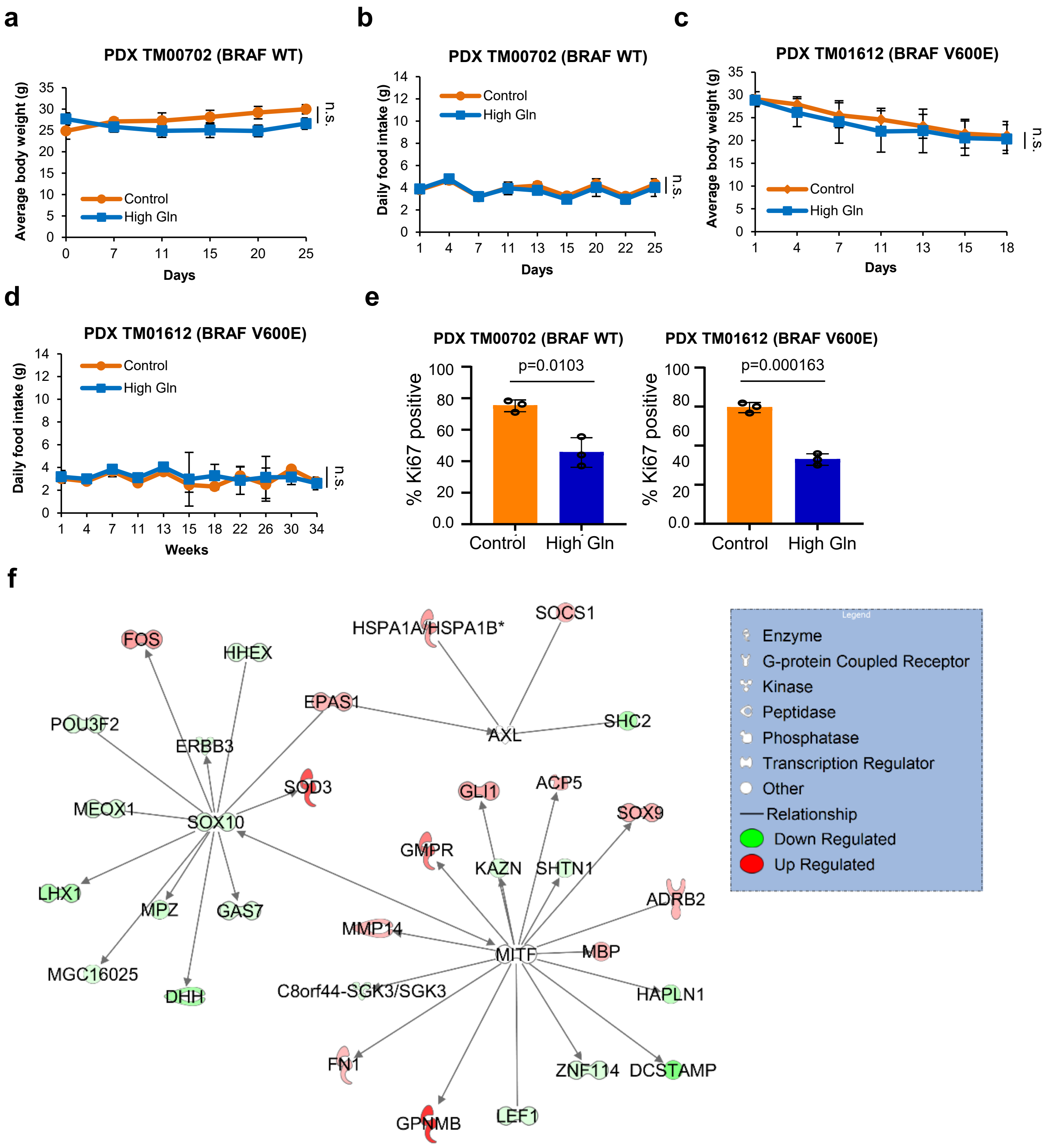

a
PDX TM00702 (BRAF WT)
Average body weight (g)
Days
Control
High Gln
n.s.
b
PDX TM00702 (BRAF WT)
Daily food intake (g)
Days
Control
High Gln
n.s.
c
PDX TM01612 (BRAF V600E)
Average body weight (g)
Days
Control
High Gln
n.s.
d
PDX TM01612 (BRAF V600E)
Daily food intake (g)
Weeks
Control
High Gln
n.s.
e
PDX TM00702 (BRAF WT)
p=0.0103
% Ki67 positive
Control
High Gln
PDX TM01612 (BRAF V600E)
p=0.000163
% Ki67 positive
Control
High Gln
f
FOS
HHEX
HSPA1A/HSPA1B*
SOCS1
EPAS1
POU3F2
ERBB3
AXL
SHC2
SOD3
MEOX1
SOX10
GLI1
ACP5
SOX9
GMPR
KAZN
SHTN1
LHX1
MPZ
GAS7
ADRB2
MMP14
MITF
MBP
MGC16025
DHH
C8orf44-SGK3/SGK3
HAPLN1
FN1
ZNF114
DCSTAMP
GPNMB
LEF1
Legend
Enzyme
G-protein Coupled Receptor
Kinase
Peptidase
Phosphatase
Transcription Regulator
Other
Relationship
Down Regulated
Up Regulated

Supplementary Figure 2. a, b. Body weight (**a**) and food intake (**b**) of NSG mice with PDX TM00702 tumours (Control, n=10; High Gln, n=9). **c, d**. Body weight (**c**) and food intake (**d**) of NSG mice with PDX TM01612 tumours (Control, n=8; High Gln, n=8). **e**. Ki67 stained microscope images of PDX TM00702 and PDX TM01612 tumours were manually quantified to determine % Ki67 positively stained cells (Control, n=3; High Gln, n=3 biologically independent tumours). **f.** Downstream signalling network of SOX10, AXL and MITF established using Ingenuity Pathway Analysis (IPA)'s Grow tool with Ingenuity Knowledge Base. 31 downstream genes of these three seed genes are significantly changed between control and high glutamine diet groups. Data represent means. Error bars are s.d. *p* value calculated by *t*-test (unpaired, two-tailed). n.s. not significant.

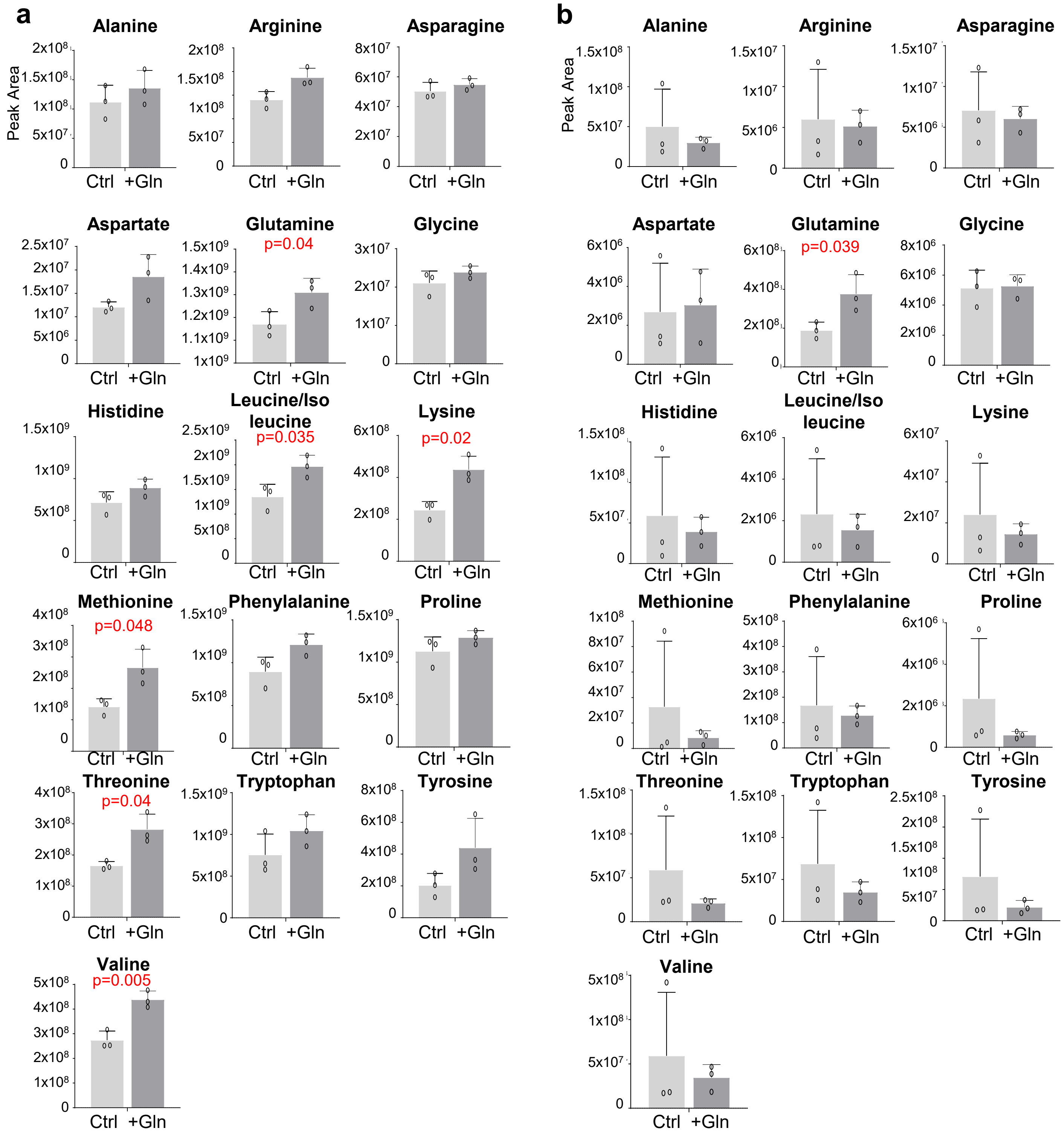

Supplementary Figure 3. NSG mice subcutaneously engrafted with PDX_TM00702 (**a**) or nude mice with M229 xenograft tumours (**b**) were placed on control (Ctrl) or glutamine supplemented (+Gln) diet and blood was collected with cardiac puncture at the end of the study. Serum was analyzed by LC-MS for relative levels of metabolites (presented as peak area on Y-axis, $n = 3$ independent biological samples). Error bars are s.d. P value calculated by t-test (unpaired, two-tailed). Only p value < 0.05 are indicated.

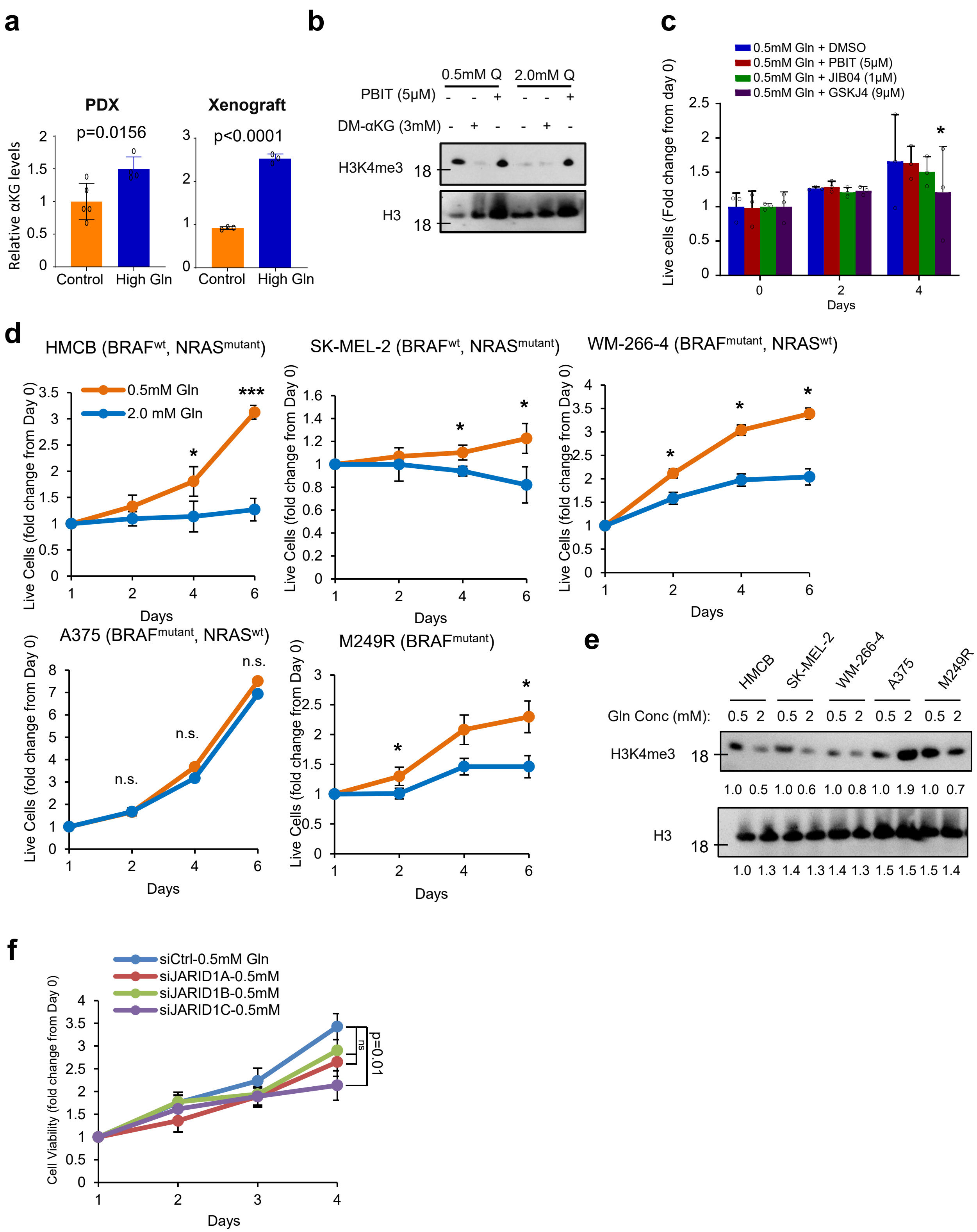

a
PDX
p=0.0156
Xenograft
p<0.0001
Relative αKG levels
Control
High Gln
b
0.5mM Q
2.0mM Q
PBIT (5μM)
DM-αKG (3mM)
H3K4me3
H3
c
0.5mM Gln + DMSO
0.5mM Gln + PBIT (5μM)
0.5mM Gln + JIB04 (1μM)
0.5mM Gln + GSKJ4 (9μM)
Live cells (Fold change from day 0)
Days
d
HMCB (BRAFwt, NRASmutant)
SK-MEL-2 (BRAFwt, NRASmutant)
WM-266-4 (BRAFmutant, NRASwt)
A375 (BRAFmutant, NRASwt)
M249R (BRAFmutant)
0.5mM Gln
2.0 mM Gln
Live Cells (fold change from Day 0)
Days
n.s.
e
HMCB
SK-MEL-2
WM-266-4
A375
M249R
Gln Conc (mM): 0.5 2 0.5 2 0.5 2 0.5 2 0.5 2
H3K4me3
1.0 0.5 1.0 0.6 1.0 0.8 1.0 1.9 1.0 0.7
H3
1.0 1.3 1.4 1.3 1.4 1.3 1.5 1.5 1.5 1.4
f
siCtrl-0.5mM Gln
siJARID1A-0.5mM
siJARID1B-0.5mM
siJARID1C-0.5mM
Cell Viability (fold change from Day 0)
Days
ns
p=0.01

Supplementary Figure 4. a. α-ketoglutarate levels were measured in PDX TM00702 and M229 xenograft tumours using EIA kit. (n=3, biologically independent tumours performed in triplicates) **b.** M229 cells culture in 6 cm dish. After 24 hours, medium was changed to either medium containing 0.5mM or 2.0mM glutamine with DMSO, PBIT (H3K4me3 demethylase inhibitor), or dimethyl-αKG. Cell lysate was used for histone extraction and immunoblotting with the indicated antibodies. The blot is representative of three independent experiments. **c.** M229 cells cultured overnight in medium containing 0.5mM glutamine under 1% hypoxia. Medium was changed after 24 hours to 0.5mM glutamine medium with DMSO, PBIT (5μM), JIB04 (1μM) or GSKJ4 (9μM), with medium changed daily. Cell viability was assessed by CellTiter Glo assay. (n=3, independent cell cultures). **d.** HMCB, SK-MEL-2, WM-266-4, A375, and M249R cells were seeded and medium was changed daily and cultured under 0.5mM or 2.0mM glutamine and live cells were counted at indicated time points (n=3, independent cell cultures). **e.** HMCB, SK-MEL-2, WM-266-4, A375, and M249R cells were seeded in 6cm dish as in **d-f**. Medium was changed daily for 4 days, and cell lysate was used for histone extraction and immunoblotting with the indicated antibodies. . The blot is representative of three independent experiments. **f.** M229 cells were transfected with siRNA against control, JARID1A, JARID1B or JARID1C and seeded in medium with 0.5mM glutamine overnight. Medium was changed daily and cultured under 0.5mM glutamine. Live cell number was assessed using Trypan blue exclusion (n=3, independent cell cultures). Data represents means. Error bars are s.d. *p* value calculated by *t*-test (unpaired, two-tailed). $^{*}p<0.05$, $^{***}p<0.001$, n.s. not significant.

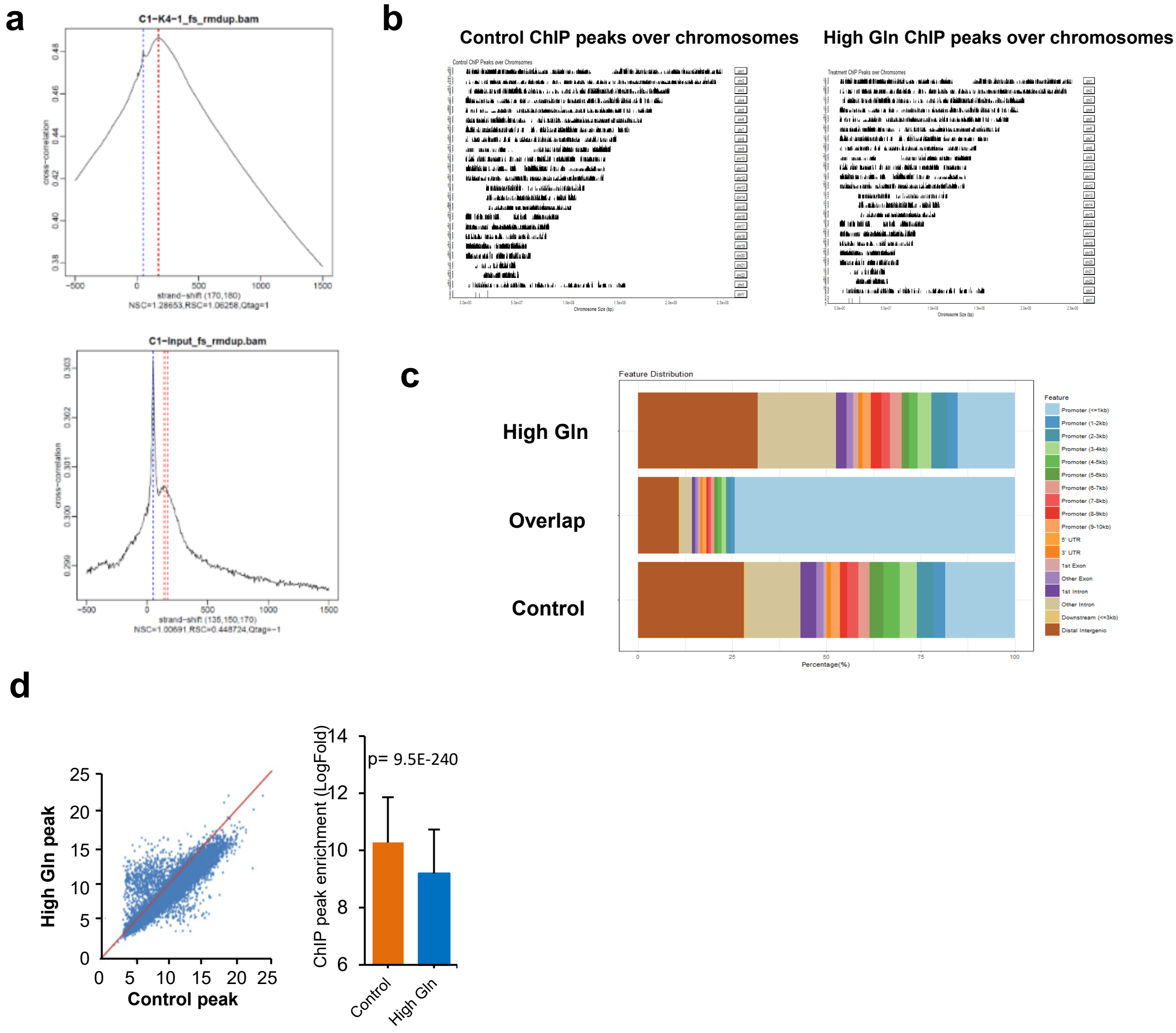

Supplementary Figure 5. a. Cross-correlation of ChIP-seq reads in sample versus sample input. One control sample and control sample input are shown as an example. **b.** ChIP-seq peaks distribution of chromosomes from control and glutamine supplemented (High Gln) samples. **c.** ChIP peaks annotation and closet genomic feature distribution in High Gln and control samples. **d.** PDX_TM00702 tumours were used for ChIP-sequencing analysis using H3K4me3 antibody. ChIP peaks of associated genes (LogFC between ChIP and Input) in control and High Gln tumour samples are represented as an average in each group (n=3, biological replicates per group performed in technical duplicates). Error bars are s.e.m. *p* value calculated by *t*-test (unpaired, two-tailed).

Supplementary Tables

Supplementary Table 1: Diets formulated for the study of glutamine supplementation in vivo, Related to Figure 1 and 2

	Control (A11112201)	High glutamine	Amino Acids
Ingredient	gm	gm	gm
L-Cystine	4.2	4.2	8.9
L-Isoleucine	7.6	7.6	16.1
L-Leucine	15.8	15.8	33.5
L-Lysine	13.2	13.2	28.0
L-Methionine	5.1	5.1	10.8
L-Phenyalanine	8.4	8.4	17.8
L-Threonine	7.2	7.2	15.2
L-Tryptophan	2.1	2.1	4.4
L-Valine	9.3	9.3	19.7
L-Histadine	4.6	4.6	9.7
L-Alanine	5.1	5.1	10.8
L-Arginine	6.0	6.0	12.7
L-Aspartic Acid	12.1	12.1	25.6
L-Glutamine	0.0	200.0	0.0
L-Glutamine Acid	38.2	38.2	80.9
Glycine	3.0	3.0	6.4
L-Proline	17.8	17.8	37.7
L-Serine	10.0	10.0	21.2
L-Tyrosine	9.2	9.2	19.5
Total Amino Acids	178.9	378.9	378.9
Corn Starch	381.0	181.0	181.0
Maltodextrin 10	110.0	110.0	110.0
Dextrose	150.0	150.0	150.0
Cellulose, BW200	75.0	75.0	75.0
Inulin	25.0	25.0	25.0
Soybean Oil	70.0	70.0	70.0
Mineral Mix	10.0	10.0	10.0
DiCalcium Phophate	13.0	13.0	13.0
Calcium Carbonate	5.5	5.5	5.5
Potassium Citrate, 1 H2O	16.5	16.5	16.5
Vitamin Mix	10.0	10.0	10.0
Choline Bitartrate	2.0	2.0	2.0
Total	1046.95	1046.95	1046.95
kcal%			
Protein	18.0	38.0	38.0
Carbohydrate	66.0	46.0	46.0
Fat	16.0	16.0	16.0
Total	100.0	100.0	100.0

Supplementary Table 2: KEGG global gene-metabolic network, related to Figure 5

Pathway	Total Cmpd	Hits	Raw p	log pvalue	FDR	Impact
Pyrimidine metabolism	47	12	1.65E-06	13.318	0.000132	0.3356
Purine metabolism	38	11	0.000636	7.3602	0.025442	0.22282
Cysteine and methionine metabolism	27	8	0.001131	6.7851	0.030146	0.44391
Amino sugar and nucleotide sugar metabolism	48	10	0.001693	6.381	0.031692	0.27581
Aminoacyl-tRNA biosynthesis	32	9	0.001981	6.2243	0.031692	0
Glutathione metabolism	41	6	0.002873	5.8525	0.03761	0.23743
Nitrogen metabolism	88	6	0.003291	5.7166	0.03761	0

p value calculated by *t*-test (unpaired, two-tailed) using MetaboAnylst
