# Supplementary material for: Dietary glutamine supplementation suppresses epigenetically-activated oncogenic pathways to inhibit melanoma tumour growth
Source: Nat Commun. 2020 Jul 3;11:3326. doi: 10.1038/s41467-020-17181-w (PMC7335172; doi:10.1038/s41467-020-17181-w)
Supplement: Supplementary file 2 — Reporting Summary [file 41467_2020_17181_MOESM2_ESM.pdf]

nature research

Corresponding author(s): Mei Kong
Last updated by author(s): May 17, 2020

Reporting Summary

Nature Research wishes to improve the reproducibility of the work that we publish. This form provides structure for consistency and transparency in reporting. For further information on Nature Research policies, see Authors & Referees and the Editorial Policy Checklist .

Statistics

For all statistical analyses, confirm that the following items are present in the figure legend, table legend, main text, or Methods section.

n/a	Confirmed	
☐	☒	The exact sample size (*n*) for each experimental group/condition, given as a discrete number and unit of measurement
☐	☒	A statement on whether measurements were taken from distinct samples or whether the same sample was measured repeatedly
☐	☒	The statistical test(s) used AND whether they are one- or two-sided<br>*Only common tests should be described solely by name; describe more complex techniques in the Methods section.*
☐	☒	A description of all covariates tested
☐	☒	A description of any assumptions or corrections, such as tests of normality and adjustment for multiple comparisons
☐	☒	A full description of the statistical parameters including central tendency (e.g. means) or other basic estimates (e.g. regression coefficient) AND variation (e.g. standard deviation) or associated estimates of uncertainty (e.g. confidence intervals)
☐	☒	For null hypothesis testing, the test statistic (e.g. *F*, *t*, *r*) with confidence intervals, effect sizes, degrees of freedom and *P* value noted<br>*Give P values as exact values whenever suitable.*
☒	☐	For Bayesian analysis, information on the choice of priors and Markov chain Monte Carlo settings
☐	☒	For hierarchical and complex designs, identification of the appropriate level for tests and full reporting of outcomes
☒	☐	Estimates of effect sizes (e.g. Cohen's *d*, Pearson's *r*), indicating how they were calculated

Our web collection on statistics for biologists contains articles on many of the points above.

Software and code

Policy information about availability of computer code

Data collection	qPCR - Bio-Rad CFX Maestro 1.0, CellTiterGlo - SoftMaxPro, LC-MS - ThermoScientific, Illumina Hiseq2500 - RTA 2.2.38.
Data analysis	Microsoft Office Excel 365 ProPlus, GraphPad Prism 7.0, RNA-sequencing HISAT2 and Partek Genome Suite (v6.6), Gene set enrichment analysis (GSEA) 3.0, Pathway analysis IMPaLA v.11, LCMS Sieve 2.0, Metaboanalyst 4.0, MSigDB database v6.2 , R’s SPP package (v1.13) , MACS2 (v2.1.2), SAMTools (ver 0.1.19), BWA (v)

For manuscripts utilizing custom algorithms or software that are central to the research but not yet described in published literature, software must be made available to editors/reviewers. We strongly encourage code deposition in a community repository (e.g. GitHub). See the Nature Research guidelines for submitting code & software for further information.

Data

Policy information about availability of data

All manuscripts must include a data availability statement. This statement should provide the following information, where applicable:
- Accession codes, unique identifiers, or web links for publicly available datasets
- A list of figures that have associated raw data
- A description of any restrictions on data availability

The authors declare that all data generated from this study are included in this publication and its supplementary information files. Data that support the findings of this study have been deposited in the Gene Expression Omnibus (GEO) under accession code GSE125822 [https://www.ncbi.nlm.nih.gov/geo/query/acc.cgi?acc=GSE125822] and GSE140274 [https://www.ncbi.nlm.nih.gov/geo/query/acc.cgi?acc=GSE140274]. The source data underlying Figs 1, 2, 4a-e, 5, 6 and Supplementary Figure 4 are provided as Supplementary Data or Source Data files. All other datasets are available from the corresponding author upon request.

Field-specific reporting

Please select the one below that is the best fit for your research. If you are not sure, read the appropriate sections before making your selection.

- [x] Life sciences
- [ ] Behavioural & social sciences
- [ ] Ecological, evolutionary & environmental sciences

For a reference copy of the document with all sections, see nature.com/documents/nr-reporting-summary-flat.pdf

Life sciences study design

All studies must disclose on these points even when the disclosure is negative.

Sample size	In vitro experiments were repeated at least three times with technical triplicates. No sample size calculations was performed for in vitro experiments, sample sizes were chosen based on experience from previous experiments to reach statistically relevant results.<br>Sample sizes for each animal study were estimated based on preliminary data or previous publication with these models predicting variance within each group (ref. 1)
Data exclusions	No mice were excluded. For B6.BRafCA, PtenloxP, Tyr::CreERT2 tri-allelic transgenic mouse, animals that died form illnesses unrelated to tumour growth were included as censored observations.<br><br>Data exclusions due to technical issues was done in these instances:<br>1. A single technical replicate was sometimes excluded from RNA-seq or LC-MS if it was clearly an outlier. Outliers were identified by Partek Genome Suite (v6.6) and MetaboAnalyst software.<br>2. One or two technical replicates were excluded from cell viability analysis if they were clearly an outlier and were one of 6 replicates in CellTiter Glo assay (identified by Microsoft excel or Graphpad Prism)
Replication	All cell culture experiments were reproduced in three independent experiments with no less than 3 replicates. For tumour analysis, replication was achieved and 3 or more biologically independent replicates were used.
Randomization	Tissue culture dishes were seeded at the same time and were randomized before treatments.<br>Animal experiments were divided randomly before changing diets or initiating treatments.
Blinding	Survival animal experiment: clinical end point was determined by a blinded animal technician.<br>IHC slides were evaluated blindly by 2 independent pathologists.<br>Cell viability was counted by a plate reader or an automated coulter counter.<br>The sample sequence of data acquisition by LC-MS was randomized.<br>Other data collection and analysis (RNA-seq and ChIP-seq) were not blinded since sample names contained treatment information.

Reporting for specific materials, systems and methods

We require information from authors about some types of materials, experimental systems and methods used in many studies. Here, indicate whether each material, system or method listed is relevant to your study. If you are not sure if a list item applies to your research, read the appropriate section before selecting a response.

Materials & experimental systems

n/a	Involved in the study	
[ ]	[x]	Antibodies
[ ]	[x]	Eukaryotic cell lines
[x]	[ ]	Palaeontology
[ ]	[x]	Animals and other organisms
[x]	[ ]	Human research participants
[x]	[ ]	Clinical data

Methods

n/a	Involved in the study	
[ ]	[x]	ChIP-seq
[x]	[ ]	Flow cytometry
[x]	[ ]	MRI-based neuroimaging

Antibodies

Antibodies used	H3K4me3 Millipore 17-614 1:2000<br>H3K9me3 Abcam ab8898 1:1000<br>H3K27me3 Millipore 17-622 1:2000<br>H3 Cell Signaling Tech 4499 monoclonal, clone# D1H2 1:2000<br>JARID1A Cell Signaling Tech 3876 monoclonal, clone# D28B10 1:1000<br>JARID1B Cell Signaling Tech 3273 1:1000<br>JARID1C Cell Signaling Tech 5361 monoclonal, clone# D29B9 1:1000<br>β-actin Sigma A1978 monoclonal, clone# AC15 1:10000

Validation	Validations are based on the datasheets from the manufacturers.

Eukaryotic cell lines

Policy information about cell lines

Cell line source(s)	Patient derived M229, M249, M249 Plx4032-resistant, M238 Plx4032-resistant cells - Dr. Roger S. Lo (UCLA)<br>HMCB, A375, SK-MEL-2, WM-266-4 - ATCC
Authentication	Cells were not authenticated.
Mycoplasma contamination	All cells were tested for mycoplasma using MycoAlert mycoplasma detection kit when first thawed.
Commonly misidentified lines<br>(See ICLAC register)	There are no commonly misidentified lines in this study.

Animals and other organisms

Policy information about studies involving animals; ARRIVE guidelines recommended for reporting animal research

Laboratory animals	NCr Nude mice (Taconic) males, 4 weeks old.<br>NOD scid gamma mice (The Jackson Lab) males, 4 weeks old.<br>B6.C57 males, 4 weeks old.<br>B6.BRafCA, PtenloxP, Tyr::CreERT2 mice (The Jackson Lab) males and females, 4 weeks old.<br>All mice were acclimated for 2 weeks and used for experiments when they are 6-weeks old. All mice were maintained under a temperature-controlled environment with a 12-hour light/dark cycle and received experimental diet and water ad libitum.
Wild animals	There are no wild animals used in this study
Field-collected samples	There are no field-collected samples used in this study
Ethics oversight	IACUC approval under University of California Irvine

Note that full information on the approval of the study protocol must also be provided in the manuscript.

ChIP-seq

Data deposition

☒ Confirm that both raw and final processed data have been deposited in a public database such as GEO.

☒ Confirm that you have deposited or provided access to graph files (e.g. BED files) for the called peaks.

Data access links<br>*May remain private before publication.*	Data that support the findings of this study have been deposited in the Gene Expression Omnibus (GEO) under accession code GSE125822 [https://www.ncbi.nlm.nih.gov/geo/query/acc.cgi?acc=GSE125822]
Files in database submission	GSM3582407 control 1 input<br>GSM3582408 control 1-1<br>GSM3582409 control 1-2<br>GSM3582410 control 2 input<br>GSM3582411 control 2-1<br>GSM3582412 control 2-2<br>GSM3582413 control 3 input<br>GSM3582414 control 3-1<br>GSM3582415 control 3-2<br>GSM3582416 treatment 1 input<br>GSM3582417 treatment 1-1<br>GSM3582418 treatment 1-2<br>GSM3582419 treatment 2 input<br>GSM3582420 treatment 2-1<br>GSM3582421 treatment 2-2<br>GSM3582422 treatment 3 input<br>GSM3582423 treatment 3-1<br>GSM3582424 treatment 3-2

Genome browser session (e.g. UCSC)

no longer applicable

Methodology

Replicates	3 independent biological tumours were used from control and glutamine groups and were run in technical duplicates and an input for each sample.
Sequencing depth	Sequencing run was performed in the single read mode of 51cycle of read1 and 7 cycles of index read using Illumina HiSeq 2500 with HiSeq SBS V4 Kits. The ChiP-Seq sequence reads were mapped to Homo sapiens genome assembly GRCh37 (hg19) using open source DNA-seq alignment tool BWA (v). High quality unique alignments were used with SAM Tag (XA:Z and SA:Z) as filter, and removed the duplicated alignments caused by PCR duplication using SAMTools (ver 0.1.19). The unmapped reads were removed using SAMTools based on the alignment quality tag (-F 4 -F 256).
Antibodies	The pull-down was performed with anti-H3K4me3 antibody (Millipore Sigma, Burlington, MA, USA).
Peak calling parameters	ChIP-seq peak calling was performed using MACS2 (v2.1.2) with broad option to detect the H3K4me3 binding sites with FDR<0.005 cutoff
Data quality	High quality unique alignments were used with SAM Tag (XA:Z and SA:Z) as filter, and removed the duplicated alignments caused by PCR duplication using SAMTools (ver 0.1.19). The unmapped reads were removed using SAMTools based on the alignment quality tag (-F 4 -F 256). The cross-correlation of the reads aligned to both strands was tested as ChiP QC using R's SPP package (v1.13) before ChIP-seq peak calling using MACS2 (v2.1.2) with broad option to detect the H3K4me3 binding sites with FDR<0.005 cutoff. The DNA fragment length is estimated based on the sequence experiment design and SPP analysis to recover the binding enrichment. ChiP peaks detected from multiple biology replications are merged to detect the highly consensus peaks. The peaks between control and high Gln samples were overlapped to detect the different enrichment using ANOVA with p<0.05 and +/- 1.5 foldchange cutoff. R's ChIPseeker package (v1.18.0) was used for ChiP peak annotation, which reports the closet genomic feature for the binding sites, feature type, and distance to TSS.
Software	The ChiP-Seq sequence reads were mapped to Homo sapiens genome assembly GRCh37 (hg19) using open source DNA-seq alignment tool BWA (v).<br>High quality unique alignments were performed using SAMTools (ver 0.1.19).<br>Cross-correlation of the reads awas tested as ChiP QC using R's SPP package (v1.13)<br>R's ChIPseeker package (v1.18.0) was used for ChiP peak annotation
